# Valorization of Anaerobic Liquid Digestates Through Membrane Processing and Struvite Recovery—The Case of Dairy Effluents

**DOI:** 10.3390/membranes15070189

**Published:** 2025-06-24

**Authors:** Anthoula C. Karanasiou, Charikleia K. Tsaridou, Dimitrios C. Sioutopoulos, Christos Tzioumaklis, Nikolaos Patsikas, Sotiris I. Patsios, Konstantinos V. Plakas, Anastasios J. Karabelas

**Affiliations:** 1 Laboratory of Natural Resources and Renewable Energies, Chemical Process and Energy Resources Institute, Centre for Research and Technology-Hellas (CERTH), 57001 Thermi-Thessaloniki, Greece; akaranasiou@certh.gr (A.C.K.); chara_t15@hotmail.com (C.K.T.); sioutop@certh.gr (D.C.S.); patsios@certh.gr (S.I.P.); kplakas@certh.gr (K.V.P.); 2 BIZIOS S.A., Vodiana, 40200 Elassona, Greece; chtziou@hotmail.com; 3 TEDRA, 23 Papanastasiou Str., 41222 Larisa, Greece; npatsikas@tedraco.com

**Keywords:** dairy industry effluents, anaerobic digestion, filtered liquid digestate valorization, nanofiltration, struvite recovery

## Abstract

An integrated process scheme is developed for valorizing filtered liquid digestates (FLD) from an industrial anaerobic digestion (AD) plant treating dairy-processing effluents with relatively low nutrient concentrations. The process scheme involves FLD treatment by nanofiltration (NF) membranes, followed by struvite recovery from the NF-retentate. An NF pilot unit (designed for this purpose) is combined with a state-of-the-art NF/RO process simulator. Validation of simulator results with pilot data enables reliable predictions required for scaling up NF systems. The NF permeate meets the standards for restricted irrigation and/or reuse. Considering the significant nutrient concentrations in the NF retentate (i.e., ~500 mg/L NH4-N, ~230 mg/L PO4-P), struvite recovery/precipitation is investigated, including determination of near-optimal processing conditions. Maximum removal of nutrients, through production of struvite-rich precipitate, is obtained at a molar ratio of NH_4_:Mg:PO_4_ = 1:1.5:1.5 and pH = 10 in the treated stream, attained through the addition of Κ_2_HPO_4_, ΜgCl_2_·6H_2_O, and NaOH. Furthermore, almost complete struvite precipitation is achieved within ~30 min, whereas precipitate/solid drying at modest/ambient temperature is appropriate to avoid struvite degradation. Under the aforementioned conditions, a significant amount of dry precipitate is obtained, i.e., ~12 g dry mass per L of treated retentate, including crystalline struvite. The approach taken and the obtained positive results provide a firm basis for further development of this integrated process scheme towards sustainable large-scale applications.

## 1. Introduction

Anaerobic digestion (AD) is a widely used wastewater treatment technology with significant advantages, such as the efficient processing of various feedstocks with high organic load and the production of biogas, which is considered a renewable energy source [1,2]. In 2021, the total number of AD/biogas plants in Europe amounted to 18,843, mainly processing feedstocks of agricultural origin, i.e., agricultural residues, sequential crops, energy crops etc. (Figure S1-1 in Supplementary Materials) [3]. However, there is also a significant potential for biogas production through AD treatment of effluents/wastes from the food processing industry, which is currently at a lower level. This study deals with the valorization of food industry digestates, which proves to be quite complicated, mainly due to their variable composition depending on AD feed conditions [4]. Therefore, the adaptation/development of appropriate processing steps is required to achieve project sustainability when such AD feedstocks are used.

The main slurry type effluent of the AD plant (i.e., the *digestate*) contains minerals, volatile fatty acids (VFAs), and nutrients (i.e., nitrogen, N, phosphorus, P, and potassium, K compounds) [5]. Primary liquid–solid separation of digestate results in two fractions: a solid (10–20% mass fraction) and a liquid (80–90%) containing the water-soluble nutrients [6]. The relatively high nutrient content of these streams has drawn attention to their valorization, especially as fertilizers, which is also in line with the new European Union (EU) Regulation 2019/1009 on Fertilizing Products [7]. The *solid fraction* is used for agricultural purposes, the production of energy, and other value-added products [8]. The *liquid digestate* (LD) can also be used for direct fertilization under controlled conditions [9,10,11]. However, in general, parameters like high salinity and other organic/inorganic micropollutants [12,13] can render LD harmful to plants and vegetation, thus hindering its direct application [14,15]. Additionally, variations in AD feedstock composition and operating parameters result in varying nutrient content [6,15,16], which should remain relatively constant when fertilization of specific plant(s) is considered. Several processes have been investigated for the treatment of LD to recover nutrients and use them efficiently in fertilizers, including ion exchange and adsorption [15,17,18], ammonia stripping [19,20], chemical precipitation [11,15], and membrane separation [10,21]. However, the development of a sustainable LD treatment scheme is still being pursued, which is challenging and requires the integration of different processes to obtain suitable fertilizer products and water for reuse or safe disposal [22,23,24].

**Figure 1:**
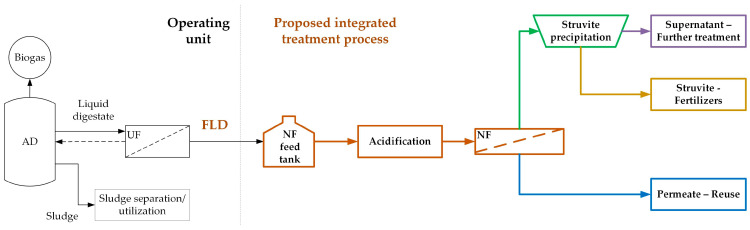
Integrated process scheme for valorization of AD filtered liquid digestate (FLD) using NF membrane treatment followed by struvite precipitation; expanded version from [29].

This paper is focused on the valorization of *dairy processing effluents*, characterized by modest concentration of organic matter and seasonal variability [25,26]. For this important type of effluent, the estimated volume produced by EU-27 industries is at the level of 200 × 10^6^ m^3^/yr [27]. Furthermore, the EU has established/listed [28] best available techniques (BAT) for treating such effluents, ensuring maximum environmental protection and sustainability. In a recent review [27] of listed BAT and other appropriate applied techniques, the need is stressed for additional R&D work to improve dairy effluent valorization. Towards this goal, an integrated system was investigated [29] aiming at the valorization of LD from an industrial AD facility (Figure 1) treating effluents from a dairy plant (“BIZIOS S.A.”) located in Thessaly, Greece. The dairy processing effluents fed to the AD unit consist mainly of cheese whey, lactose, “flushings”, and CIP (Cleaning in Place) water from the production lines. The liquid digestate from the AD unit is treated with ultrafiltration (UF) membranes (Memthane^®^ technology from Veolia Water Technologies, Delft, The Netherlands [30]) to remove suspended solids (SS), whereas the excess sludge is separated through centrifugation. In the investigated scheme [29] (Figure 1), the produced filtered liquid digestate (FLD, i.e., the UF permeate), free from most of its original organic load, is further treated by nanofiltration (NF) membranes to effectively retain the dissolved inorganic species in the concentrate. However, due to the significant FLD alkalinity, a pretreatment step prior to NF is necessary [29], i.e., controlled acidification to reduce alkalinity to mitigate NF membrane scaling. The NF permeate stream is suitable for industrial reuse or restricted irrigation [31], while the concentrate can be utilized for nutrient recovery. Other relevant studies [32,33] have shown that NF treatment can play a critical role by concentrating phosphate-containing effluents, leading to sufficient saturation of phosphate salts and a reduction in the chemicals needed for their crystallization/recovery. This study focuses on the recovery of nutrients in the form of struvite from such NF concentrates.

The precipitation of magnesium ammonium phosphate (MgNH_4_PO_4_·6H_2_O, MAP), an effective fertilizer commonly known as struvite [34,35,36,37,38], has been efficiently used for the simultaneous recovery of nutrients (i.e., ammonium and phosphate ions). In fact, industrial systems are already in operation, with annual production of MAP reaching ~78 kt in the EU-28 [39,40,41]. Nevertheless, there are significant knowledge gaps, as process performance is influenced by several parameters, including pH [42,43,44], duration of precipitation [45], the molar ratio of struvite components in the feed solution (NH_4_:Mg:PO_4_) [44,46,47], magnesium (Mg) source [45,46,48], and the composition of the effluent [49,50,51]. The effects of these parameters on struvite precipitation have been extensively studied using mostly synthetic solutions [48,52,53,54]. However, the efficiency of the process should be carefully evaluated using real LD samples in order to obtain realistic design data for such plants. Moreover, investigations have been reported on dairy industry waste-streams with *high* nutrient content [55,56]. Yet, the recovery of nutrients from wastewaters with rather *low* nutrient concentration (of interest to this study) remains a challenge that should be addressed [22,23,24].

Literature studies along the lines indicated above are briefly outlined. Lopez et al. [57] evaluated the feasibility of struvite precipitation from a NF retentate stream using a synthetic solution that simulated the secondary effluent of a wastewater treatment plant. Through experiments and simulations, they concluded that 70% permeate removal resulted in adequate concentration for struvite precipitation without Mg addition. Nir et al. [32] investigated the recovery of phosphorous from low P concentration effluents through continuous NF and calcium phosphate crystallization. Arola et al. [33] applied a two-stage NF treatment to the effluent of a membrane bioreactor treating municipal wastewater. The final concentrate stream was fed into a settling tank to precipitate phosphates in the form of hydroxyapatite. Ramaswami et al. [34] investigated the recovery of ammonia from raw methanogenic landfill leachates and different streams from membrane processes through struvite precipitation.

In the aforementioned study by Tsaridou et al. [29], a comprehensive methodology was applied that included bench and cross-flow tests with synthetic and real FLD, as well as reliable simulations to determine the key design parameters of an NF pilot plant. Such a pilot unit was initially tested with synthetic solutions. In the present study, the performance of an appropriate NF pilot unit is tested in an industrial environment to optimize the concentration of FLD, also taking into account FLD variability. Furthermore, the valorization of the NF concentrate through struvite precipitation is systematically investigated. The effects of key parameters, including pH, duration of struvite precipitation, molar ratio of NH_4_:Mg:PO_4_ in the feed solution, and Mg source, on process performance are investigated. Such a systematic investigation of the key process parameters for struvite recovery from *real effluents* is rarely found in the literature, although it is essential to draw clear conclusions regarding process sustainability.

## 2. Struvite Formation Reaction and Process Performance Indicators

Struvite is formed through the following chemical reaction:

Mg^2+^ + NH_4_^+^ + H_n_PO_4_^n−3^ + 6H_2_O → MgNH_4_PO_4_·6H_2_O + (n + 1)H^+^ (1)

In principle, the stoichiometric ratio of Mg^2+^, NH_4_^+^, and PO_4_^3−^ (1:1:1) is required to form struvite, and (depending on the FLD composition) phosphorous and/or magnesium must be added to the AD effluent to achieve the desired supersaturation conditions [48]. In addition, pH is a key factor in the formation of struvite [58]. Therefore, pH adjustment of the feed solution was investigated in the alkaline range, as it has been repeatedly reported that optimal pH values are in the range of pH 8.0–10.0 [23,59,60].

To evaluate struvite precipitation from a given feed solution, the removal ratio (R_i_) [61,62] of each ionic component (i.e., Mg^2+^, NH_4_^+^ and PO_4_^3−^) is determined as follows:

(2) $$R_{i}=\frac{C_{b_{i}}-C_{f_{i}}}{C_{b_{i}}}$$

where *C_bi_* and *C_fi_* are the concentrations of component i in the initial solution *after the addition of the Mg^*2*+^ and PO_*4*_*^3−^ ions and in the final solution (after precipitation), respectively. This parameter, which is expressed as the percentage removal efficiency (i.e., %*R_i_*), is a useful indicator for an effective comparison between feed solutions with different compositions of Mg^2+^ and PO_4_^3−^ ions.

To assess the quality of the formed precipitate, the struvite purity (%) is determined according to Cui et al. [63] using the following expression:

(3) $$Struvite\;purity\;\%=\frac{C_{N}\cdot \frac{V}{M_{N}}\cdot M_{MAP}}{m_{MAP}}\cdot 100$$

where *C_N_* (mg/L) is the concentration of NH_4_-N in the HCl solution after dissolution of the precipitate, *V* (L) is the volume of the HCl solution, *M_N_* and *M_MAP_* in g/mol are the molar masses of nitrogen and struvite, respectively, and *m_MAP_* is the mass of the recovered precipitate.

## 3. Materials and Methods

### 3.1. Filtered Liquid Digestate (FLD)

The FLD used in this study was obtained from the AD plant (operating at BIZIOS S.A dairy industry) after filtration through UF membranes (Figure 1). Based on the analyses of different samples of this effluent (Table S2-1 in the Supplementary Materials), it is evident that there are significant variations of the main physicochemical parameters, including electrical conductivity (eC: 6100–8300 μS/cm), total alkalinity (2400–3700 mg CaCO_3_/L), sodium (1400–1750 mg/L), and chloride ion (300–800 mg/L) concentrations. The aforementioned variations are attributed to significant differences in the composition of the AD feedstock due to seasonality but also the different operating conditions of the industrial AD bioreactor. Due to the significant FLD alkalinity, acidification prior to NF was considered necessary. It should also be noted that the FLD samples used in the pilot tests had a higher concentration of mainly inorganic and, in some cases, organic species compared to the samples used in lab-scale tests of our previous study [29]. The samples (in batches of 250, 500, and 1000 L) from the FLD were firstly treated in the NF pilot unit, and the concentrates were subsequently used for the chemical precipitation tests for struvite recovery.

### 3.2. Nanofiltration (NF) Pilot Unit Tests

The design of the NF pilot unit, based on a systematic methodology that included both laboratory experiments and reliable computer simulations, was reported by Tsaridou et al. [29]. This unit is equipped with two pressure vessels (Phoenix ltd, Gloucester, MA, USA) in series. Each pressure vessel contains two NF90-2540 (DuPont™ FilmTec™, Wilmington, DE, USA) spiral wound membrane (SWM) modules. The effective membrane area of each module is 2.6 m^2^, so that the total membrane area per vessel is 5.2 m^2^. Three tanks (1000 L each) are used to store the feed, concentrate, and permeate streams, whereas systems/controls for pH adjustment and CIP are also installed. The feed stream is pressurized with a multistage horizontal pump (1HM22N11T, LOWARA, Xylem Inc., Washington, DC, USA). The pilot NF unit can be operated in different modes (i.e., batch, continuous, and semi-continuous) depending on the requirements of the particular experimental test protocol.

**Figure 2:**
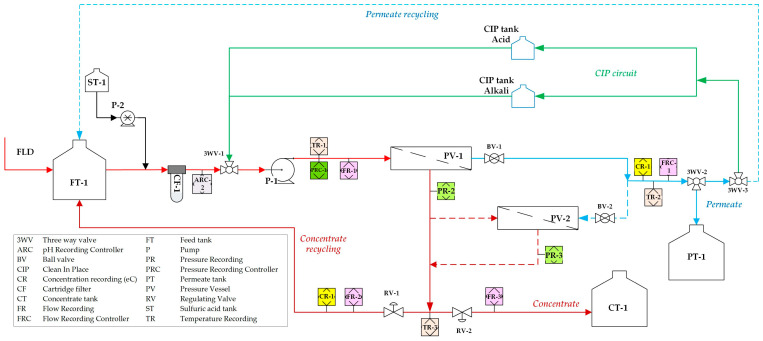
Process flow diagram of the NF pilot unit employed in this study.

The pilot unit is fully controlled by a programmable logic controller (XBC/XEC U-Type, LS ELECTRIC Co., Ltd., Anyang-si, Republic of Korea). A human–machine interface with a touch screen (MT8090XE, WEINTEK, New Taipei City, Taiwan) is used for monitoring and data acquisition (SCADA, Supervisory Control And Data Acquisition). Remote control of the unit is possible from any electronic device connected to the Internet, e.g., from a computer (desktop or laptop), a tablet, or a smartphone using the RealVNC^®^ Viewer 7.10.0 application. The operating parameters of the pilot unit and the specifications of the membrane given by the manufacturer [64,65,66] are listed in Table A1 in Appendix A. The pilot unit was installed in the premises of the BIZIOS S.A. dairy processing plant (Figure 2).

Prior to the pilot tests, pretreatment of the FLD feed with sulfuric acid (6N) was necessary to reduce its alkalinity, which affects the performance of the NF membranes [29]; these samples were designated as AFLD (acidified FLD). The coding of the experiments in the present work is given in Table A2 in Appendix A. Initially, two tests (Pilot-1_NFOT and Pilot-2_NFOT) were performed with the pretreated FLD (by recirculating both NF permeate and retentate, thus simulating the *once-through* flow mode) to assess the performance of the pilot unit compared to the computer simulations. Next, five NF pilot tests (Pilot3_NFBM to Pilot5_NFBM and Pilot-6_NFBM-SP to Pilot-7_NFBM-SP) were performed in *batch mode* to concentrate AFLD by continuously recycling the retentate to the feed tank while collecting the permeate stream. After pilot tests Pilot-6_NFBM-SP and Pilot-7_NFBM-SP, which were conducted in batch mode, concentrated AFLD samples were collected (i.e., NFC-6 and NFC-7, Table 1), which were used for struvite recovery. Considering the size of the pilot plant, the test conditions were adjusted for an initial permeate recovery rate of ~13% per pass. Specifically, the feed flow rate was set to 15 L/min (corresponding to a typical cross-flow velocity of 0.25 m/s), and the permeate flow rate was set to 2 L/min. The system applied pressure that varied between 9 and 10 bar. The variation of the applied pressure was due to the different ionic strength of the AFLD used in each test, which affected its osmotic pressure. At the end of each batch pilot test, the quantity of the collected permeate corresponded to a permeate recovery of 50 ± 10%. The factors hindering greater NF permeate recovery included the significant flux reduction due to the increase of the feed TDS concentration and osmotic pressure, the undesirable precipitation of salts, and membrane scaling phenomena, as explained below. A membrane cleaning protocol was applied between tests, as described in the Supplementary Materials (Section S3-1).

### 3.3. Solutions and Chemical Reagents for Struvite Recovery Tests

Two different samples of NF concentrate streams (NFC-6 and NFC-7) from AFLD were used as feed solutions for the struvite recovery tests. Their physicochemical characteristics are summarized in Table 1. Overall, the chemical composition of the NFC streams (in terms of nutrients concentration) is quite similar to other feed water streams employed for struvite recovery [59,67]. Table 1 (from both retentate samples) shows that the ammonium ions (NH_4_^+^) are in significant molar excess compared to the PO_4_^3−^ and Mg^2+^ ions with respect to the struvite molecular structure (MgNH_4_PO_4_·6H_2_O). For instance, the molar ratio of Mg:NH_4_:PO_4_ in NFC-6 is approx. 1:14:3. Therefore, the addition of phosphates and magnesium ions is necessary to achieve the desired equimolar ratio (1:1:1) for maximum struvite precipitation.

The chemical reagents used in the struvite precipitation tests were anhydrous potassium phosphate dibasic (Κ_2_HPO_4_ ≥ 99.0%, Honeywell International Inc., Charlotte, NC, USA), magnesium chloride hexahydrate (MgCl_2_·6H_2_O 99.0–102.0%, PanReac AppliChem, ITW Reagents srl, Milano, Italy), magnesium oxide (MgO 100.1%, Lach-Ner S.R.O., Neratovice, Czech Republic), and sodium hydroxide (NaOH ≥ 99.0%, Riedel-de-Häen^TM^, Honeywell International Inc., Charlotte, NC, USA). Two commercial MgO grades were also used, the “Emag45” grade (95%, Sibelco Group, Antwerp, Belgium) and a fast-calcined grade (98.6%, Calix Ltd., Pymble, Australia). The former (MgO-C1) is produced conventionally and has a low specific surface area (BET = 32 m^2^/g), while the latter (MgO-C2) exhibits a substantially higher BET (188–232 m^2^/g). MgO is practically insoluble in water [68], but it is assumed that MgO-C2 produces a homogeneous suspension due to its high BET.

### 3.4. Struvite Precipitation Tests

A laboratory study with NF concentrate (NFC-6) as feed water was performed using a JarTest system (AMF4, I.S.Co srl, Bologna, Italy), which is described in Section S3-2 of the Supplementary Materials. The aim was to determine a near-optimal range of operating parameters leading to maximum recovery of nutrients from the aforementioned NF retentate of an FLD stream, considering also process sustainability issues. The struvite precipitation protocol, the detailed experimental conditions, and some informative pictures of the lab-scale tests can be found in the Supplementary Materials, Section S3-3, Table S3-1, and Figure S3-1, respectively.

The parameters examined in the bench-scale tests were the pH, ranging from 8 to 11, the liquid/solid separation method, the drying temperature of the precipitate (~25 °C/ambient, 40 °C and 105 °C), the NH_4_:Mg:PO_4_ molar ratio in the feed solution, and the Mg^2+^ source (MgCl_2_·6H_2_O and MgO). Finally, for validation purposes, a realistic test was performed with a large amount of feed (100L NFC7) under near-optimal conditions, defined in the preceding bench-scale tests (precipitation test PS-13_100L).

### 3.5. Analytical Methods

Detailed analyses were performed for all treated solutions (FLD, AFLD, NFC, NFP, precipitation supernatant, SPS, and filtrate, SPF) to determine their main physicochemical characteristics: pH (744 pH Meter, Metrohm AG, Herisau Switzerland), eC (Multi 3510 IDS, WTW, Xylem Analytics GmbH & Co, Weilheim, Germany), ion concentration (IC—Ion Chromatograph, Prominence Series, Shimadzu, Kyoto, Japan), TOC (TOC-L CSN analyzer with TNM-L, Shimadzu Co., Kyoto, Japan), elemental analysis (ICP—Inductively Coupled Plasma unit, Optical Emission Spectrometer, Model Optima 4300 Dv, PerkinElmer, Waltham, MA, USA), and total alkalinity (automatic titrator, 877 Titrino Plus, Metrohm AG, Herisau, Switzerland). The precipitate was characterized using a scanning electron microscope (SEM, JSM-IT500LV, Jeol, Tokyo, Japan) coupled with an Energy Dispersive X-ray Spectrometry unit (EDS Χ-ACT, Oxford Instruments, Abingdon, UK) for simultaneous elemental analysis. Additionally, the crystalline form was determined through X-Ray Diffraction (XRD, D500, Siemens, Munich, Germany). Finally, a certain amount of precipitate was dissolved in 0.06N HCl (Honeywell International Inc., Charlotte, NC, USA) solution for elemental and nutrient analyses with ICP and IC. The saturation index SI of the process solutions was determined with the computer program PHREEQC (version 3.7.3.15968) by implementing the minteq.v4 database [69].

## 4. Experimental Results

### 4.1. Nanofiltration (NF) Pilot Tests

All pilot tests were conducted using only one pressure vessel (Figure 2) including two spiral wound NF modules with a total membrane area of 5.2 m^2^. Permeate recovery was set to 13.3% per pass, i.e., the feed flow was controlled to 15 L/min and the permeate flow to ~2 L/min. Recovery was set in accordance with the manufacturer’s requirements regarding the feed characteristics [65]. The detailed test conditions are listed in Table S3-2 in the Supplementary Materials. It should also be noted that the FLD employed in these tests had higher ionic strength compared to that used in the laboratory experiments presented in a previous publication [29].

#### 4.1.1. Once-Through Mode

The first series of tests (Pilot-1_NFOT and Pilot-2_NFOT), designed to assess the performance of the pilot unit compared to computer simulations, was essentially carried out in a “once-through” flow mode by maintaining constant feed conditions. This was achieved by employing a fairly large (500 and 1000 L) feed fluid volume and recirculating both NF permeate and retentate streams. The composition of the feed stream, the concentrate, and the permeate, as well as the rejection values, are summarized in the Supplementary Materials (Table S4-1). A pristine membrane was used in the “Pilot-1_NFOT” test, while mild chemical cleaning of the system was performed prior to the Pilot-2_NFOT test (described in Section S3-1 of the Supplementary Materials).

**Figure 3:**
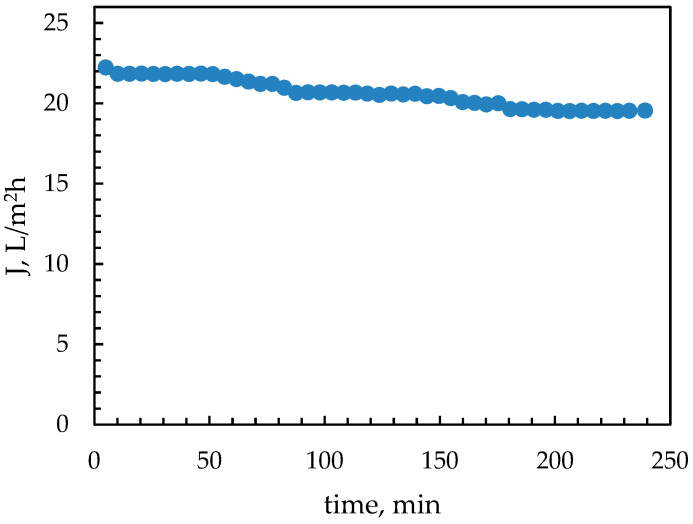
Measured flux profile in test Pilot-2_NFOT.

Figure 3 depicts the temporal variation of the mean permeate flux during the Pilot-2_NFOT test. The reported mean flux was determined by dividing the mean permeate flow rate by the total membrane active surface. The flux exhibited a small decline during the test due to membrane fouling and scaling phenomena, which affected its permeability. However, as shown in the diagram, these phenomena are limited due to the relatively low permeate recovery (~13%) prevailing in these tests. Therefore, for the tested permeate recovery, it is considered that the NF system operates at a steady state, and a meaningful comparison can be made with reliable NF system simulations. For this purpose, special software was employed (designated as SWM/NRRE), developed in the authors’ laboratory for simulating the performance of RO/NF SWM elements at a steady state [70,71,72]. This state-of-the-art simulator, based on background theoretical work, CFD simulations, and experiments, takes into account all relevant phenomena, including concentration polarization. The software simulates the flow field throughout the membrane sheets, and the spatial distribution of all process parameters can be predicted. For the reported simulations, an effective permeability of 5.6 L/m^2^·h·bar was employed, which is typical of a used membrane after chemical CIP [29]. Design parameters of the pilot unit, and specifications of the NF-90 membrane SWM element used, are listed in Table A1. The input parameters for the simulations reported here are included in Table A3 of Appendix A.

**Figure 4:**
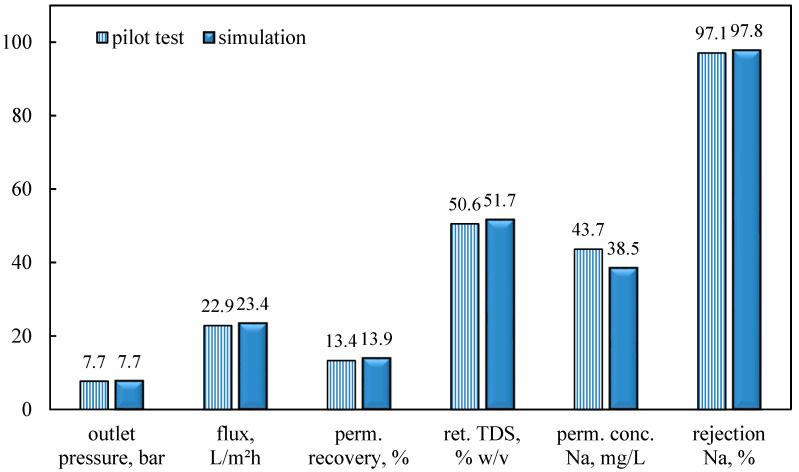
Comparison of pilot test data with simulation results for test ‘Pilot-2_NFOT’.

The results of simulations are compared with the main experimental key performance parameters of the pilot tests, i.e., the pressure at the outlet of the pressure vessel, the mean flux and permeate recovery, the TDS concentration of the retentate at the outlet of the pressure vessel, the mean sodium concentration of the permeate at the outlet of the pressure vessel, and the mean sodium rejection. The input data for the simulations correspond to the typical once-through test ‘Pilot-2_NFOT’. The membrane module design and operating parameters, input into the Simulator NRRE/SWM and corresponding to Pilot-2_NFOT test, are listed in Table A3. As shown in Figure 4, the experimental data are in fair agreement with model predictions. The relatively small differences may be attributed to some organic fouling and the increasing concentration of ionic species along the membrane modules (resulting in incipient membrane scaling), which was not taken into account in the simulations.

**Figure 5:**
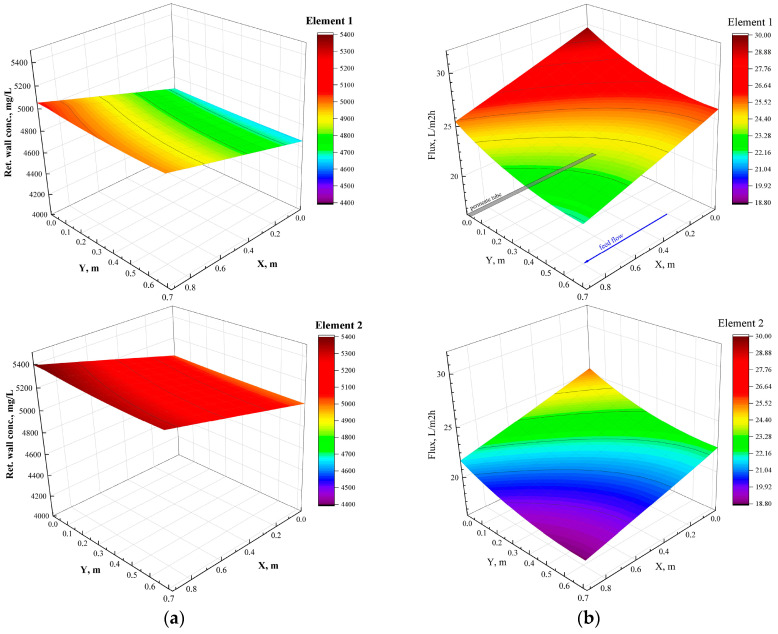
Spatial variation on SWM membrane sheets of (**a**) wall concentration/TDS on the membrane and (**b**) permeate flux in 1st and 2nd SWM elements of the pilot unit. Simulation results corresponding to conditions of test ‘Pilot-2_NFOT’. The arrow designates the mean flow direction.

Figure 5 offers useful insights, showing typical detailed simulation results corresponding to those depicted in Figure 4. The diagrams of Figure 5a exhibit a clear increase in the concentration of the ionic species along the membrane surface due to permeate removal. Thus, the local concentration towards the exit of element #2 is the highest and may exceed the supersaturation limits (depending on feed and other conditions), thus leading to incipient scaling, e.g., [73]. As is also well-known [74], the spatial wall–concentration distribution is closely associated with the spatial variation of local flux, as shown in Figure 5b, i.e., as the wall concentration increases in the axial X direction, osmotic pressure also increases, and the local trans-membrane pressure decreases, leading to significantly decreasing flux. In summary, the fair agreement between NF pilot data and related simulation results provides assurance that reliable predictions and design of a large-scale NF installation are possible by combining pilot test results with computer simulations, taking into account the specific properties of the feedstock.

#### 4.1.2. Batch Mode

**Figure 6:**
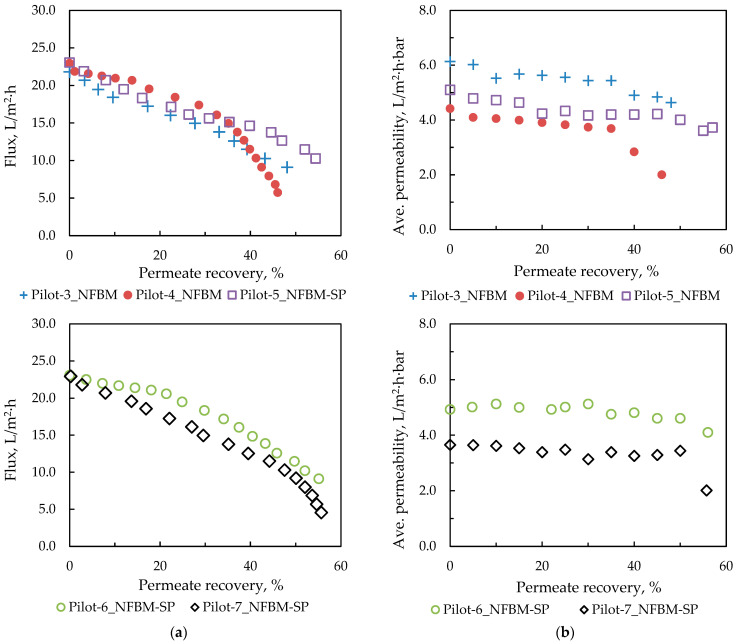
(**a**) Permeate flux and (**b**) average permeability profiles during batch NF tests; the significant steady decline of flux with recovery (panels a) is due to the strong effect of increasing osmotic pressure. The concentrates of tests Pilot-6 NFBM-SP and Pilot-7 NFBM-SP were used for struvite precipitation tests.

A second set of pilot tests was conducted by recirculating the concentrate to the feed vessel and collecting the permeate in a separate vessel until a total permeate recovery of 50 ± 10% was achieved. The detailed test conditions are summarized in Table S3-2 in the Supplementary Materials. It should be noted that the inlet pressure was controlled/adjusted due to variations in feed concentration to achieve an initial permeate recovery of 13.3% per pass. Figure 6a shows the flux profiles of the batch pilot tests. A smooth flux decline is observed at permeate recovery up to ~40%, attributed to the increasing osmotic pressure of the effluent entering the unit. However, at a permeate recovery of more than ~50%, a sharper flux decline is observed, which is likely due to membrane scaling. A clearer picture of the results can be obtained by calculating the average temporal membrane permeability *Kp_ave_* (in L/m^2^·h·bar) according to the following equation:

(4) $$K_{p_{ave}}=\frac{J}{{\Delta Ρ}_{ave}-{\Delta \pi }_{ave}}$$

where *J* is the permeate flux (in L/m^2^h) and (Δ*P_ave_* − Δ*π_ave_*) is the average effective trans-membrane pressure taking into account the applied pressure and the TDS of the effluent at the inlet and outlet of the pressure vessel. The effective osmotic pressure difference *Δπ* is expressed as the difference of osmotic pressure of the feed solution at the membrane surface minus the permeate osmotic pressure. Osmotic pressure is calculated using computational routines [75,76] for the thermophysical properties of seawater, which are given as functions of temperature, pressure, and TDS. The TDS of the feed solution is calculated considering the species concentration *C_w,i_* at the membrane surface based on concentration polarization phenomena [77]:

(5) $$\frac{C_{w,\;i}}{C_{b,i}}=\left(1-R_{i}\right)+R_{i}\cdot exp(\frac{J}{k_{i}})$$

where *C_w,I_* and *C_b,I_* are the ion concentrations at the membrane surface and in the bulk solution, respectively, and *R_i_* and *J* are the measured ion rejection and permeate flux, respectively. The mass transfer coefficient *k_i_* was calculated using a reliable correlation of the dependence of Sherwood on the Reynolds and Schmidt number developed by Koutsou et al. [78]. As shown in Figure 6b, the initial average permeability of a relatively fresh membrane after chemical CIP (Pilot 3_NFBM, initial feed volume 500 L) is 6.1 L/m^2^·h·bar. In contrast, when the membrane is subjected to simple flushing (Pilot 4_NFBM, initial feed volume 1000 L), the initial average permeability is significantly lower (4.4 L/m^2^·h·bar) and exhibits an early sharp drop at permeate recovery > 40% [Figure 6b]; this is due to the deposits on the membrane that have formed during the prolonged operation of the pilot unit. In the two subsequent tests (Pilot-5_NFBM and Pilot-6_NFBM-SP, initial feed volume 250 L), the initial average permeability was about 5.0 L/m^2^·h·bar, because they were subjected to an intensive chemical CIP and the feed solutions had similar concentrations, as listed in Table S4-2 in the Supplementary Materials (eC 7530 and 8250 mS/cm, alkalinity 2546 and 2408 mg CaCO_3_/L, respectively). The feed solution to the Pilot-7_NFBM-SP test exhibited the highest total alkalinity (3283 mg/L CaCO_3_) and longer (double) duration, which was due to the larger initial feed volume (500 L). Therefore, the membrane in this test exhibited lower initial permeability caused by the membrane scaling, resulting in a sharp decrease at >50% recovery. In fact, the concentrate (at 50% permeate recovery) was found to exceed the saturation limits for various calcium, phosphate, and carbonate salts by employing the PHREEQC (version 3.7.3.15968) computer program to estimate the respective saturation indices. Specifically, the saturation indices (SI) for calcite, dolomite (disordered), dolomite (ordered), hydroxyapatite, and Ca_3_(PO_4_)_2_(beta) were greater than 0 (0.08, 0.29, 0.84, 7.37 and 0.79, respectively), indicating their precipitation tendency. The concentrates from the Pilot-6 NFBM-SP and Pilot-7 NFBM-SP tests were used for the struvite precipitation tests.

**Figure 7:**
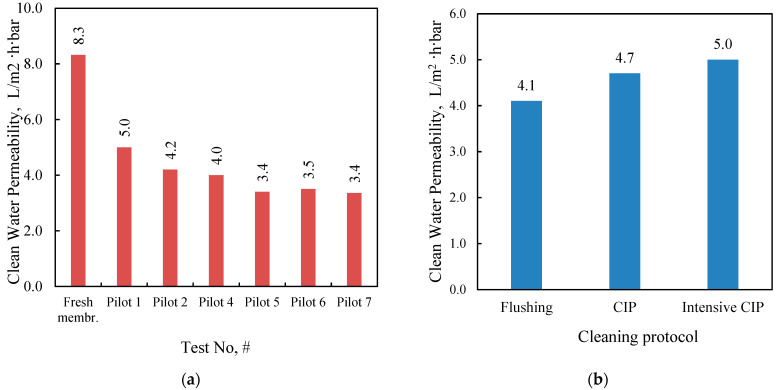
Membrane average clean water permeability (**a**) after each test and after flushing the membrane; (**b**) after test No Pilot-4_NFBM and after applying the indicated cleaning protocol; constant flux approx. 28–30 L/m^2^h.

Clean water tests were carried out between the NF pilot tests and after the application of the cleaning protocols (described in detail in the Supplementary Materials). Tap water was used for flushing the NF pilot unit, preparing the CIP solutions, and performing the clean water tests. The suitability of the tap water for this purpose was verified through physicochemical analyses (eC: 681 µS/cm, TOC: 0.47 mg/L, and negligible alkalinity). In tests with clean (tap) water, the feed flow was set to 15–16 L/min and the flux to 28–30 L/m^2^h. Figure 7a shows the permeability of the pristine membrane (determined by Equation (4)) and after the membrane tests. It should be noted that a cleaning process (through flushing) of the membrane was performed after each test. The relatively low clean water permeability of the NF membrane even after membrane flushing necessitated chemical CIP. As depicted in Figure 7b, the applied cleaning protocol has a significant effect on membrane permeability. In particular, intensive CIP seems to have a positive effect on membrane permeability but does not lead to its complete restoration. An intensive CIP procedure was carried out prior to pilot tests no. 5, 6, and 7.

Regarding the prevailing membrane fouling/scaling mechanism and related effects, the new data should be interpreted in connection with the more detailed fouling data obtained in the previous/companion study [29]. The latter was performed (with FLD and the same NF90 membrane) at bench scale. These data show that in addition to the significant inorganic scaling, a thin organic fouling layer tends to cover the membrane (e.g., Figure 7 and Table 2 in [29]). Previous studies (e.g., [77]) suggest that organic fouling layers may enhance scaling. The new data are in line with the previous results [29], showing that even with fairly intensive chemical CIP protocols, total restoration of membrane permeability is impossible. This partial permeability restoration can be attributed to irreversible membrane fouling, likely due to pore blockage. In summary, the new data obtained under realistic conditions at pilot scale provide useful insights into the cleaning, lifetime, and replacement frequency of SWM elements in a full-scale NF installation.

**Figure 8:**
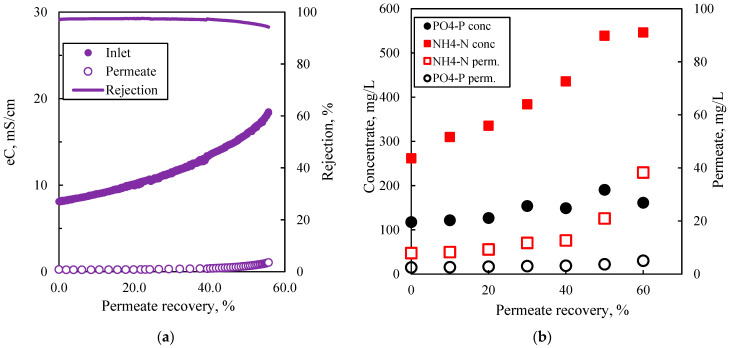
Effect of permeate recovery on (**a**) electrical conductivity, (**b**) nutrient concentration in the concentrate; Test No Pilot-7_NFBM-SP, inlet pressure 11 bar, feed flow ~15 L/min (u~0.25 m/s).

The last two NF pilot runs in batch mode (i.e., Pilot-6_NFBM-SP and Pilot-7_NFBM-SP) were performed with permeate recovery of 56% to obtain a sufficiently concentrated volume required for the subsequent struvite precipitation process. Figure 8a shows that the membrane exhibited high ion rejection (>94%) in terms of electrical conductivity when permeate recovery reached approximately 60%. Furthermore, it can be seen that AFLD was about twice as concentrated after NF treatment, except for orthophosphate ions, which decreased significantly at >50% permeate recovery, likely due to supersaturation and precipitation of phosphate salts, as shown in Figure 8b.

The detailed characteristics of the feed, the final concentrate, and the cumulative permeate are listed in Table 1.

### 4.2. Results of Struvite Precipitation Tests

The process of struvite precipitation was investigated considering the concentration of struvite building blocks (i.e., NH_4_^+^, PO_4_^3−^, Mg^2+^) in the feed solution, in the supernatant (before or after filtration), and in the precipitate. The most important process parameters investigated in this study were the pH, the duration of struvite precipitation, the molar ratio NH_4_:Mg:PO_4_ in the feed solution, and the magnesium source. The experimental conditions of the tests are listed in Table S3-1 in the Supplementary Materials.

#### 4.2.1. The Effect of pH

Samples from the concentrate of test Pilot-6_NFBM-SP (sample NFC-6) were used for the parametric study of struvite recovery using the jar testing method. First, the effect of pH (equal to 8, 9, 10, and 11) on the removal efficiency of each nutrient was determined at equimolar concentrations of Mg^2+^, NH_4_^+^, and PO_4_^3−^ (1:1:1), which was achieved by the addition of ortho-phosphate and magnesium ions. As expected, an increase in pH is beneficial for the precipitation of struvite and the removal of Mg^2+^, determined by Equation (2). This can be attributed to the competitive behavior of Ca^2+^ and Mg^2+^ in the formation of different types of phosphate salts [79]. Furthermore, a slight increase in NH_4_^+^ removal (up to ~69%) was observed at pH 11, which is probably due to the removal of ammonia in gaseous form at high pH values [60]. Increasing the pH from 10 to 11 appears to have a negligible effect on Mg^2+^ removal; thus, all subsequent precipitation tests were performed at pH 10, considering the increased operating cost by adding NaOH for a rather small increase in struvite precipitation. At the molar ratio tested, the NH_4_^+^ concentration in the filtered supernatant solution (SPF) exceeds the limits for safe disposal or reuse as reclaimed water. Indeed, according to national legislation [31], the TAN (Total Ammonium Nitrogen) limit is 45 m/L for restricted irrigation, industrial use, and recharge of certain underground aquifers in areas that are not sensitive to nitrate pollution, whereas the concentration of TAN in SPF is 150–200 mg/L. Thus, the ammonia concentration in the supernatant should be further reduced for safe disposal. This issue was addressed by adding Mg and PO_4_ salts, as described in Section 4.2.3. Detailed data related to this sub-section (Section 4.2.1) are included in Section S4-2.1 of the Supplementary Materials.

#### 4.2.2. Duration of Struvite Precipitation Process

**Figure 9:**
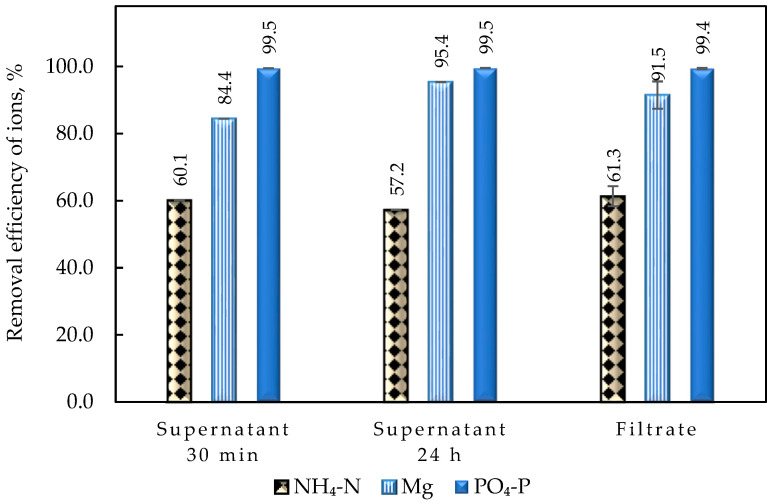
Effect of precipitate separation method on NH_4_-N, Mg, and PO_4_-P and removal; Mg source: MgCl_2_·6H_2_O, feed solution NH_4_:Mg:PO_4_ = 1:1:1; pH = 10 (test No SP-3).

The concentration of NH_4_-N and PO_4_-P in the solution after struvite precipitation was determined after (a) 30 min and (b) 24 h of settling and (c) filtration of the supernatant solution with a 1.5 μm glass fiber filter after 24 h to evaluate the kinetics/evolution of struvite precipitation and the efficiency of filtration to recover the precipitate compared to settling. As seen in Figure 9, no significant differences in ionic species removal were observed between a precipitation time of 30 min and 24 h, indicating that solids’ precipitation is almost complete within half an hour, and no significant change in struvite precipitation is observed thereafter. Specifically, ammonium removal at pH 10 remained at ~60%, while for phosphate it was >99%, in both cases (i.e., 30 min and 24 h). The results of this study are in complete agreement with the findings of previous studies [45], demonstrating that Mg, NH_4_, and PO_4_ complexation leads to struvite precipitation within a 30 min period. As expected, the comparison of the concentrations in the supernatant solution (SPS) with the corresponding values of the filtrate (SPF) shows that there is no significant difference in the determined removal efficiency of the ionic struvite components (Table S4-4 in the Supplementary Materials). The small decrease in Mg removal for SPF can be attributed to the dissolution of Mg salts due to agitation during filtration. These observations suggest that an additional separation process (and/or filtration) is not needed to efficiently recover the solid precipitate. Nevertheless, it should be emphasized that NH_4_-N removal remains low (~60%) in all previously described tests. Therefore, the effect of the molar ratio NH_4_:Mg:PO_4_ in the bulk solution on the recovery of struvite was also investigated, as discussed subsequently (Section 4.2.3).

#### 4.2.3. Effect of the NH_4_:Mg:PO_4_ Molar Ratio

**Figure 10:**
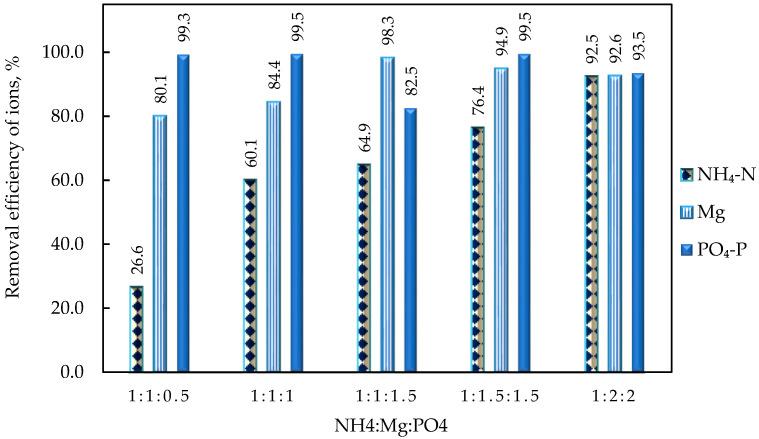
Effect of NH_4_:Mg:PO_4_ molar ratio on removal of struvite ionic components; pH = 10, 30 min of precipitation (test Nos SP-3, SP-5, SP-6, SP-7, SP-8).

As mentioned in an earlier section (Section 4.2.1), the NH_4_^+^ concentration in the supernatant after struvite precipitation remains substantially high (195–255 mg/L) at equimolar concentrations of NH_4_, Mg, and PO_4_ in the feed solution. Therefore, the effect of the NH_4_:Mg:PO_4_ molar ratio in the bulk solution on the removal of nutrients through struvite precipitation was investigated to determine the optimum ratio that would maximize the recovery of these nutrients. It is noted that the desired molar ratio was achieved by adding the appropriate amount of Κ_2_HPO_4_ and ΜgCl_2_·6H_2_O in the feed solution. All tests were performed at a pH of 10 to achieve greater recovery of struvite precipitate and lower operating costs (by minimizing the added NaOH solution), as described in Section 4.2.1. Figure 10 shows the removal of struvite building blocks for all tested molar ratios based on the concentration values in the supernatant solution after precipitation (SPS) and settling for 30 min. This duration is considered sufficient for complete precipitation of the struvite according to the results of the previous section (Section 4.2.2).

The results show that in the case of an equimolar ratio of NH_4_ and Mg^2+^ (1:1), increasing PO_4_^3−^ (in the range of 0.5 to 1.5) steadily increased the removal of magnesium ions from ~76% to ~98%. Moreover, an increase in ΝH_4_^+^ binding from ~27% το ~65% is also observed, resulting in a NH_4_-N concentration in the supernatant (30min of precipitation) of ~180 mg/L, as shown in Table S4-5 of the Supplementary Materials (test with a molar ratio NH_4_:Mg:PO_4_ equal to 1:1:1.5). This result suggests that the concentrations of Mg^2+^ and PO_4_^3−^ should be further increased to reduce the NH_4_^+^ concentration and maximize struvite recovery. Indeed, NH_4_:Mg:PO_4_ molar ratios of (1:1:1.5) to (1:1.5:1.5) and (1:2:2) resulted in an increase in ammonium removal from 65% to 76.4% and 92.5%, respectively.

The results show that the ortho-phosphate ions were completely removed from the feed solution in almost all molar ratios, indicating their complete complexation with the available cations (Mg^2+^, Ca^2+^, and NH_4_^+^). However, increasing PO_4_^3−^ ions above the equimolar ratio to Mg^2+^ (i.e., NH_4_:Mg:PO_4_ equal to 1:1:1.5) resulted in decreased ortho-phosphate removal (82.5%); this is attributed to the excess of phosphate ions in the feed solution that could not be further complexed with the available struvite structural blocks. The simultaneous increase of Mg^2+^ and PO_4_^3−^ ions in the feed solution led to a restoration of the percentage of phosphate removal to values > 93%. However, an increased excess of Mg^2+^ and PO_4_^3+^ (i.e., NH_4_:Mg:PO_4_ equal to 1:2:2) resulted in a slight decrease in the removal efficiency of Mg and PO_4_-P, showing that they cannot further precipitate as struvite.

The total amounts of dry struvite produced at different NH_4_:Mg:PO_4_ molar ratios are summarized in Table 2. In agreement with the percentage removal of the struvite ionic components (Figure 10), it can be observed that increasing the molar ratio of Mg^2+^ and PO_4_^3−^ leads to an increased precipitate mass per unit volume of bulk solution. Dissolution tests of the solid precipitate indicate that at the higher molar ratios (i.e., 1:1.5:1.5 and 1:2:2) there is no significant difference in the nutrient concentration in the precipitate, apart from a slight increase in precipitate mass. SEM and XRD analyses were also carried out on all precipitates after drying at ambient temperature (Figures S4-4 and S4-5 in the Supplementary Materials, respectively). The formed crystals, as depicted in the SEM images, exhibit polyhedral, hopper, and rough (dendritic) structures, which is indicative of struvite [42,51]. The XRD spectra show that the crystalline form of struvite dominates.

As shown in Table S4-5 in the Supplementary Materials, although the maximum removal of NH_4_ (94%) was achieved at a molar ratio of NH_4_:Mg:PO_4_ of 1:2:2, the final concentration of PO_4_ ions in the supernatant (30 min) was quite high (446 mg/L), despite the high removal efficiency (93.5%). Therefore, it can be concluded that the precipitation of phosphate ions reaches a plateau above a certain molar ratio, despite the availability of NH_4_^+^ (48 mg/L) and Mg^2+^ (129 mg/L) ions in the feed solution. The analysis of the dissolved precipitate of the NH_4_:Mg:PO_4_ 1:2:2 test supports the assertion above, as the N, Mg, and P content (2.4%, 11%, and 12.7%, respectively) is quite similar to that of the NH_4_:Mg:PO_4_ 1:1.5:1.5 test (N: 2.9%; Mg: 10.7%; and P: 11.8%).

#### 4.2.4. Effect of the Drying Temperature on the Precipitate’s Structure

To investigate the effect of the drying procedure on the struvite’s structure, the separated solid precipitate (test No SP-3: equimolar ratio 1:1:1, pH 10) was dried at three different temperatures, namely, room temperature (~25 °C), 40 °C, and 105 °C, and the moisture content was determined based on the weight difference of the precipitate before and after the drying process. During this procedure, the weight loss of the dry precipitate was observed by increasing the temperature, which is in accord with the literature and similar data [63] and was attributed to the release of ammonia and crystallized water during drying. Consequently, it can be assumed that water molecules and ammonia evaporate during the drying process, as also suggested by Sutiyono et al. [80]. The structural instability of struvite at elevated temperatures has also been reported in the literature (e.g., [51,81]). According to Guan et al. [51], this can be attributed to the thermal decomposition of struvite to ammonium nitrogen or amorphous magnesium sodium phosphate. Therefore, it can be concluded that a drying process at temperatures up to 40 °C should be selected for the struvite precipitate to avoid degradation of its structure. The detailed results of this part of the study are described in Section S4-2.4 of the Supplementary Materials.

#### 4.2.5. Effect of the Mg^2+^ Source

**Figure 11:**
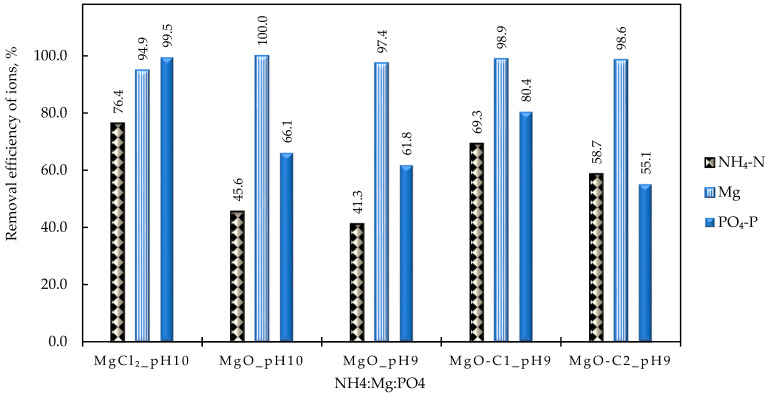
Effect of the Mg source on the removal of struvite components; NH_4_:Mg:PO_4_ 1:1.5:1.5, MgCl_2_: analytical grade MgCl_2_·6H_2_O; MgO: analytical grade MgO; MgO-C1: commercial MgO with low BET; MgO-C2: commercial MgO with high BET (test Nos SP-7 and SP-9 to SP-12).

The type of magnesium source added to the feed solution to create favorable conditions for struvite precipitation significantly affects the process’s efficiency [22]. As described in Section 3.3, three different types of MgO sources were used in this study, namely, analytical grade, MgO-*C1*, and MgO-*C2*, instead of MgCl_2_·6H_2_O, for a given molar ratio NH_4_:Mg:PO_4_ (i.e., 1:1.5:1.5). It should be noted that the addition of MgO led to an increase in the pH of the initial solution to ~9.0. When analytical grade MgO was used, the pH was adjusted to 10 by adding NaOH (test No SP-9). However, no pH adjustment was made in the next tests (SP-10 to SP-12). As seen in Figure 11, the use of MgO results in an almost complete removal of Mg^2+^ ions from the feed solution. However, the removal of PO_4_^3−^ and NH_4_^+^ was significantly lower (66.1 and 45.6, respectively) than in the case of MgCl_2_ (99.5 and 76.4, respectively). Furthermore, increasing the pH from 9 to 10, in the case of the analytical grade MgO, had a negligible effect on the removal of the three struvite structural components. Finally, it is emphasized that the high BET commercial MgO (MgO-C2) showed an insignificant increase in ion removal compared to the low BET commercial MgO (MgO-C1). However, it should be noted that both reagents were more effective in terms of ion removal than the analytical grade one. The lower removal observed with MgO compared to MgCl_2_ can be attributed to the low solubility of MgO in water, suggesting that the available amount of Mg ions is significantly lower than expected, resulting in limited removal of PO_4_^3−^ and NH_4_^+^ ions. Information on the struvite components before and after 30 min of precipitation can be found in Table S4-7 in the Supplementary Materials.

**Figure 12:**
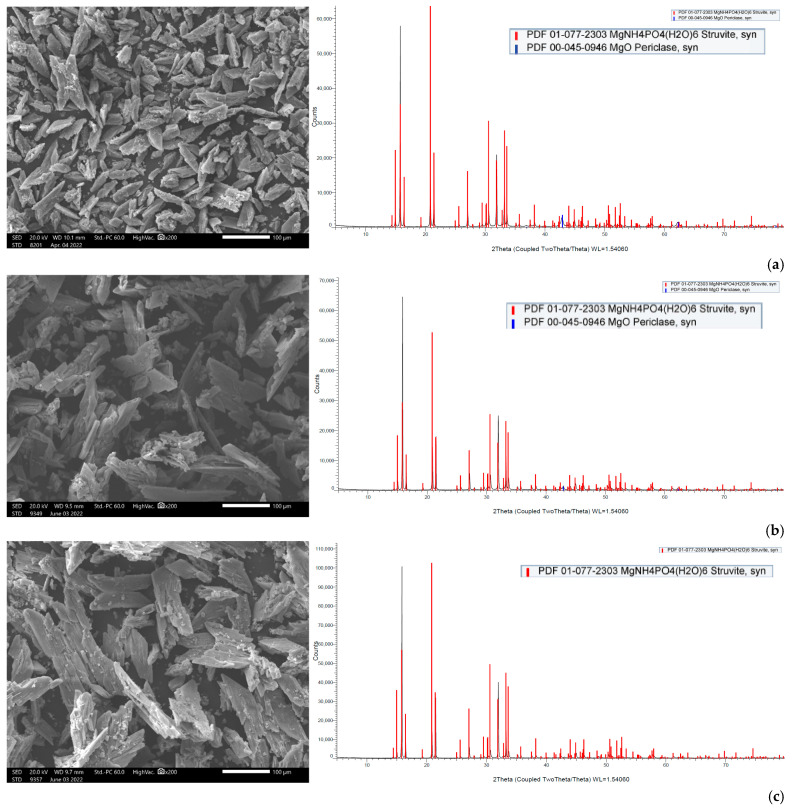
SEM images and XRD spectrum of dry precipitate after precipitation with (**a**) analytical grade MgO, (**b**) MgO-C1, (**c**) MgO-C2; red peaks: struvite; blue peaks: periclase (test Nos SP-10 to SP-12).

The suggestion of partial dissolution of the MgO reagents is supported by the increased Mg concentration in the precipitate (Table S4-8 in the Supplementary Materials). Additionally, as shown in Figure 12, through XRD analysis, MgO in the form of *periclase* (peaks in blue) is identified, whereas the SEM images show that the size of the precipitated inorganic crystals tends to increase in the case of commercial MgO.

Summarizing the above results, it can be concluded that MgO could be a possible alternative as a Mg^2+^ source for struvite precipitation, as it enhances the desirable solution alkalinity and thus reduces the cost/need for NaOH addition. However, its low solubility is a significant limitation to its use in practice.

#### 4.2.6. Large-Scale Precipitation Test

After investigating the effects of the main process parameters on the chemical precipitation of struvite, it is suggested that the near-optimal conditions for the efficient treatment of NF retentate include the following values: pH 10, precipitation time 30 min, drying of the precipitate at ambient temperature, use of MgCl_2_·6H_2_O as the main source of Mg^2+^, and a molar ratio of NH_4_:Mg:PO_4_ 1:1.5:1.5 in the feed solution.

Based on the above conditions, a large-scale test (test No SP-13_100L) was conducted to recover struvite using 100 L of NFC solution from the industrial digestate (Pilot-7_NFBM-SP, Table 1). In addition, solid Κ_2_HPO_4_ and ΜgCl_2_ 6H_2_O were used for struvite precipitation (to adjust the molar ratio in the feed solution to 1:1.5:1.5) and NaOH solution (5N) for pH adjustment. The precipitate was dried overnight at room temperature after complete settling of the solids and removal of the supernatant. Both the supernatant solution (30 min after precipitation) and the precipitate (after dissolution with 0.06N HCl) were characterized physicochemically. The results are listed in Table 3.

The physicochemical analysis of the effluent after struvite precipitation (Table 3) clearly suggests that the supernatant effluent could be safely disposed of or reused only after additional treatment to reduce conductivity, alkalinity, sodium ion, and nutrient concentrations.

**Figure 13:**
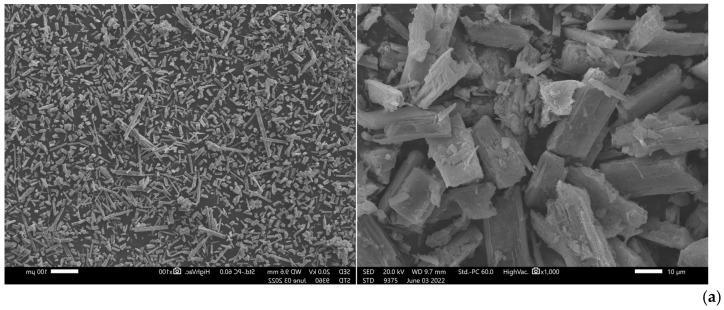

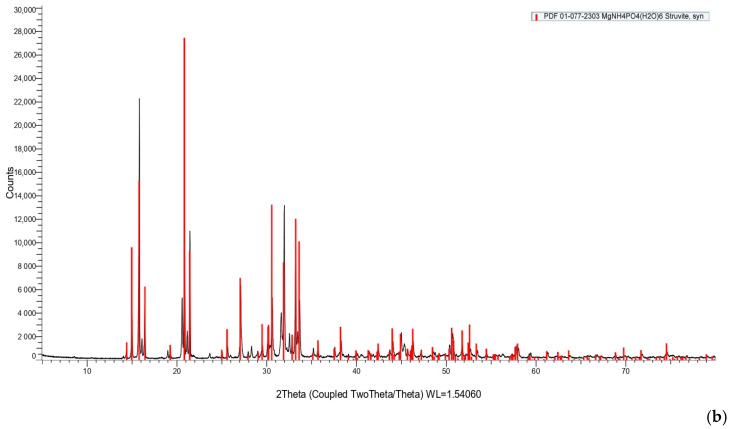
(**a**) SEM images (×100 and ×1000) and (**b**) XRD spectrum (black line) of the dried sample of the precipitation test with a molar ratio of ΝH_4_:Mg:PO_4_ 1:1.5:1.5 at a large scale (100 L) using MgCl_2_·6H_2_O; pH = 10, precipitation time = 30 min; red line representing pure struvite spectrum.

The mass of the produced solid precipitate from the 100 L pilot test (test No SP-13_100L), was 11.0 g per L of feed solution on a dry basis, which is consistent with the results of the corresponding bench-scale tests, suggesting that the struvite precipitation process is efficient and scalable (Table 2). Moreover, the elemental analysis of the dry precipitate is similar to that of the lab tests. From the elemental composition of the precipitate, shown in Table 3, it can be concluded that it is suitable for soil fertilization in terms of phosphorus content [58]. It is also interesting to note that the molar ratio of Mg and PO_4_ in the *precipitate* (NH_4_:Mg:PO_4_ 1:2:2) is greater than that of the *feed solution after the addition of K_*2*_HPO_*4*_ and MgCl_*2*_·6H_*2*_O* (NH_4_:Mg:PO_4_ 1:1.5:1.5). Based on the XRD analysis of the final precipitate’s structure in Figure 13, the excess of Mg^2+^ and PO_4_^3−^ ions in the precipitate can be attributed to the formation of other amorphous species (likely magnesium phosphate) in coexistence with struvite.

## 5. Discussion

In this study, an integrated method for the valorization of the liquid digestate of an AD plant is investigated. The methodology includes first the concentration of industrial FLD with an NF pilot unit and the subsequent recovery of nutrients from the concentrate stream through struvite precipitation. The results of the nanofiltration pilot tests have shown that for this particular FLD, permeate recovery of 50 ± 10% and a concentration factor of nutrients ~2 is feasible, i.e., under controlled membrane scaling. An acidification processing step (before NF) was necessary to control the increased and variable FLD alkalinity, which would lead to undesirable salt precipitation and membrane scaling. Conditions for maintaining/restoring the NF membrane’s permeability through periodic cleaning (flushing, CIP) have been investigated at pilot scale. An advanced NF process simulator, whose satisfactory performance was confirmed through comparison with the realistic pilot data, can be used to assist process scale-up and control. The NF permeate cannot be disposed of in sensitive receiving water bodies as the NH_4_-N disposal limits (≤2 mg/L) are not met. However, it can be safely used for industrial purposes and/or restricted irrigation [31]. Unrestricted irrigation is also an option after appropriate dilution.

Particular attention was paid to the parametric study of struvite precipitation of the NF concentrate stream, as it contains valuable nutrients, which are the main target of this work. Therefore, a systematic investigation of the key operating parameters for higher precipitation efficiency was carried out. In all cases, the addition of a PO_4_^3−^ and Mg^2+^ source was necessary due to the excess of NH_4_^+^ in the liquid digestate. Magnesium chloride proved to be more effective, even though the addition of NaOH was necessary to adjust the pH. The increased pH values favor the formation of struvite compared to other phosphate precipitates and contribute to increased Mg^2+^ binding. Thus, pH 10 was selected for treatment of the specific NF concentrate, taking into account the operating costs. The concentration of the three building blocks of struvite (NH_4_^+^, Mg^2+^, PO_4_^3−^) in the supernatant remains almost constant 30 min after the start of struvite precipitation and over a period of approximately 24 h. Therefore, it can be concluded that the ion complexation for struvite formation occurs quite quickly, and chemical precipitation is practically completed within 30 min. Drying the precipitate at high temperature (105 °C) leads to the dissociation of struvite, as confirmed through XRD analysis. In contrast, at lower drying temperatures (25 °C and 40 °C), the crystalline form of struvite was clearly identified. Therefore, it is suggested that the precipitate should be dried at ambient temperature. To improve ammonium removal through struvite formation, the addition of Mg^2+^ and PO_4_^3−^ salts in excess is necessary. A near-optimal ΝH_4_^+^:Mg^2+^:PO_4_^3−^ molar ratio of 1:1.5:1.5 was determined experimentally.

Under the aforementioned near-optimal operating conditions, the following removal efficiencies were achieved: 76.4% for NH_4_^+^, 94.9% for Mg^2+^, and 99.5% for PO_4_^3−^. The mass of dry struvite produced was ~11.7 g/L of the NF concentrate’s volume, consisting of ~3% *w*/*w* ΝH_4_-N, ~11% Mg^2+^, and 12% *w*/*w* PO_4_^−^P. Although the concentration of nutrients in the struvite precipitate does not meet the minimum content specified in the EU regulation for commercial use [7], it could be utilized in the production of fertilizers or directly on selected crops, depending on the nutrient content and ratio. The reproducibility of the results for struvite recovery obtained in the laboratory and on a larger scale is considered satisfactory for future applications on a pilot/industrial scale. Furthermore, because the recovered struvite originates from controlled dairy industry effluents, the potential fertilizer produced would be free of heavy metals, emerging contaminants, etc. Finally, the inorganic load of the supernatant solution/effluent resulting from the precipitation process is still quite high (despite the lower nutrient content) for direct disposal. Therefore, this effluent should be treated further before reuse or disposal.

Finally, it is emphasized that the compositional variability, the relatively high conductivity, and the alkalinity of the liquid digestate are important issues in the management of agro-industrial effluents by AD. Therefore, combined appropriate operations (such as those presented in this study) are necessary to overcome such issues and achieve a sustainable process for FLD valorization.

## 6. Conclusions

The approach to valorize FLD demonstrated herein involves two stages:

1. *NF membrane treatment* to concentrate FLD under near-optimal conditions (i.e., to reach the maximum possible concentration level without causing irreversible membrane scaling and undesirable salt precipitation) depending on the (usually varying) FLD properties. An NF pilot unit (designed for this purpose) is combined with a state-of-the-art NF/RO process simulator. Validation of simulator results with experimental data enables reliable simulations/predictions required for scaling up NF systems.
2. *Struvite recovery* from the NF concentrate through conditioning, controlled precipitation/separation, and solids drying. Near-optimal conditions for maximizing the recovery of nutrients (P, N, Mg) in the form of struvite are experimentally determined, including pH, the molar ratio of NH_4_:Mg:PO_4_, solids’ precipitation time, and drying temperature.

The effluent/permeate from the first stage, approx. 50% of the FLD treated here, can be used for restricted irrigation or reuse, whereas the effluent/supernatant from the second stage requires additional treatment for disposal or reuse due to the significant ionic strength.

The approach and integrated processing scheme for FLD valorization demonstrated in this study at pilot scale for typical dairy AD effluents enables the collection of reliable data needed for a sustainability assessment of similar industrial-scale projects, as well as for monitoring/controlling similar operating facilities under varying feed properties.

## Figures and Tables

**Table 1:** Physicochemical characteristics of feed, retentate, and permeate streams from tests Pilot-6_NFBM-SP and Pilot-7_NFBM-SP.

	Pilot-6_NFBM-SP			Pilot-7_NFBM-SP		
Parameter	Feed AFLD-6	Concentrate NFC-6	Permeate NFP-6	Feed AFLD-7	Concentrate NFC-7	Permeate NFP-7
pH	6.7	7.1 ± 0.1	5.7	7.3	7.4	5.8
eC, μS/cm	9380	18,350 ± 75	736	9670	18,800	465
Mg^2+^, mg/L	27	61 ± 7 (2.5 mM)	1.4	34	44 (1.8 mM)	1.0
NH_4_-N, mg/L	242	509 ± 20 (36 mM)	16.2	262	546 (38.7 mM)	14.1
PO_4_-P, mg/L	106	247 ± 15 (8 mM)	4.6	118	161 (5.2 mM)	15.4
Cl^−^, mg/L	662	1533 ± 145	45.0	997	1318	33.2
SO_4_^2−^, mg/L	1401	4230 ± 90	46.9	599	2950	19.7
Na^+^, mg/L	1894	4083 ± 81	135	1942	3322	78.9
K^+^, mg/L	254	545 ± 35	18.0	258	618	13.2
Ca^2+^, mg/L	18.5	33 ± 8	1.6	ND	26	<2
Alkalinity, mg CaCO_3_/L	2408	3440 ± 30	258	3283	4612	181.2
TOC, mg/L	139	274	15	NA	118	5.8

AFLD-6: acidified FLD-III; AFLD-7: acidified FLD-V; NFC-6 at permeate recovery: 55.1%; NFC-7 at permeate recovery: 55.7%; NA: not analyzed, ND: not detected.

**Table 2:** Dry mass and nutrients concentration in the precipitate (test Nos SP-3, SP-6, SP-7, SP-8).

ΝH_4_:Mg:PO_4_	1:1:1	1:1:1.5	1:1.5:1.5	1:2:2
Mass of dry precipitate per feed volume, g/L	8.2	14.0	11.8	16.3
NH_4_-N, % *w*/*w* ^db^	4.4	1.9	2.9	2.4
Mg^2+^, % *w*/*w* ^db^	11.0	6.9	10.7	11.0
PO_4_-P, % *w*/*w* ^db^	13.0	11.0	11.8	12.7
ΝH_4_:Mg:PO_4_ in precipitate ^db^	1:1.4:1.3	1.0:2.1:2.7	1:2.1:1.9	1:2.6:2.3

^db^ dry basis.

**Table 3:** Physicochemical characteristics of the precipitate and supernatant solution of the pilot test; NH_4_:Mg:PO_4_ molar ratio of 1:1.5:1.5 (test No SP-13_100L).

Precipitate ^db^	Supernatant, 30 min
TN: 2.9 %wt	ΝH4-Ν: 174 mg/L
Mg: 9.9 %wt	PO4-P: 299 mg/L
TP: 12.6 %wt (ICP)	Mg^2+^: 116 mg/L
K: 5.0 %wt	K^+^:4025 mg/L
Ca: 0.8 %wt	Na^+^:6543 mg/L
Zn: 7.6 mg/kg	Cl^−^: 4799 mg/L
Fe: 14.4 mg/kg	SO_4_^2−^: 3424 mg/L
pH: 9.9 (1:1000 *v*/*v*)	Alkalinity: 7304 mg CaCO_3_/L
eC: 163 mS/cm (1:1000 *v*/*v*)	pH:10.1
NH4: Mg:PO_4_ 1:2:2 (ICP)	eC:32.6 mS/cm

^db^ dry basis.

## Data Availability

The data presented in this study are available upon request from the corresponding authors.

## Supplementary Materials

The following supporting information can be downloaded at https://www.mdpi.com/article/10.3390/membranes15070189/s1.

## Abbreviations

The following abbreviations are used in this manuscript:

AD	Anaerobic digestion
AFLD	Acidified filtered liquid digestate
BET	Brunauer–Emmett–Teller
CIP	Cleaning in Place
eC	Electrical conductivity
EDS	Energy Dispersive X-ray Spectrometry
FLD	Filtered liquid digestate
IC	Ion chromatograph
ICP	Inductively coupled plasma
LD	Liquid digestate
MAP	Magnesium ammonium phosphate (MgNH_4_PO_4_·6H_2_O)
MgCl_2_	Analytical grade MgCl_2_·6H_2_O
MgO	Analytical grade MgO
MgO-C1	Commercial MgO with low BET
MgO-C2	Commercial MgO with high BET
NF	Nanofiltration
NFC	Nanofiltration concentrate
NFP	Nanofiltration permeate
PLC	Programmable logic controller
SCADA	Supervisory control and data acquisition
SEM	Scanning electron microscope
SP	Struvite precipitation test
SPF	Struvite precipitation filtrate
SPS	Struvite precipitation supernatant
SWM	Spiral wound membrane
TOC	Total organic carbon
UF	Ultrafiltration
VFA	Volatile fatty acids

## Appendix A

**Table A1:** Design parameters of the pilot unit and specifications of the NF-90 membrane elements.

Pilot Design Parameters	
No of SWM modules	4
Operating temperature	30 ± 5 °C
Operating feed flow rate	15 ± 2 L/min
Operating permeate flow rate	2 ± 2 L/min
Operating pressure	10 ± 5 bar
Concentrate eC range	0–20 mS/cm
Permeate eC range	0–2000 μS/cm
Operating pH	7 ± 2
Cleaning pH	1–12
Membrane Properties	
Material	Membrane: Polyamide Thin-Film Composite/Outer wrap: tape
Element type	2540
Active area	2.6 m^2^
MWCO	200 Da
Permeate flow ^1^, L/min	2.6 m^3^/d (1.8 L/min) −20%/+30%
Minimum salt rejection ^1^	97.0%
Operating pH ^2^/short-term cleaning pH	2–11/1–13
Maximum operating pressure	41 bar
Maximum operating temperature	45 °C
Maximum feed flow rate	1.4 m^3^/h (23.3 L/min)
Maximum Pressure Drop	0.9 bar
Pilot Design Parameters	
No of SWM modules	4
Operating temperature	30 ± 5 °C
Operating feed flow rate	15 ± 2 L/min
Operating permeate flow rate	2 ± 2 L/min
Operating pressure	10 ± 5 bar
Concentrate eC range	0–20 mS/cm
Permeate eC range	0–2000 μS/cm
Operating pH	7 ± 2
Cleaning pH	1–12
Membrane Properties	
Material	Membrane: Polyamide Thin-Film Composite/Outer wrap: tape
Element type	2540
Active area	2.6 m^2^
MWCO	200 Da
Permeate flow ^1^, L/min	2.6 m^3^/d (1.8 L/min) −20%/+30%
Minimum salt rejection ^1^	97.0%
Operating pH ^2^/short-term cleaning pH	2–11/1–13
Maximum operating pressure	41 bar
Maximum operating temperature	45 °C
Maximum feed flow rate	1.4 m^3^/h (23.3 L/min)
Maximum Pressure Drop	0.9 bar

^1^ Test conditions: 2,000 ppm MgSO_4_, 25 °C at 15% recovery at 4.8 bar. ^2^ Continuous operation at pH > 10 for maximum temperature of 35 °C.

**Table A2:** Coding of the experiments.

NF Pilot Test Code	Type of Test	FLD, #	Struvite Precipitation Test Code
Pilot-1_NFOT	Pilot NF once-through	I	-
Pilot-2_NFOT	Pilot NF once-through	II	-
Pilot-3_NFBM	Pilot NF batch	I	-
Pilot-4_NFBM	Pilot NF batch	II	-
Pilot-5_NFBM	Pilot NF batch	NA	-
Pilot-6_NFBM-SP	Pilot NF batch–struvite precipitation	III	SP-1 to SP-12
Pilot-7_NFBM-SP	Pilot NF batch–struvite precipitation	V	SP-13

NA: Not Applicable

**Table A3:** Module design and operating parameters for the Simulator NRRE/SWM, corresponding to Pilot-2_NFOT test.

*Feed Solution Characteristics*	
Feed solution TDS, kg/m^3^	4464
*Operating parameters*	
Feed flow rate, L/min	14.8
Inlet pressure, bar	8.65
*Module design parameters*	
Membrane resistance *, m^−1^	77.7·10^13^
Number of envelopes	2
Membrane sheet length	0.92 m
Membrane sheet width	0.71 m
Gap of feed channel	0.7 mm
Gap of permeate channel	0.22 mm
Feed spacer filament diameter	0.35 mm
Number of elements	2

* Corresponding to effective permeability of 5.6 L/m^2^·h·bar.
