# Supplementary material for: Valorization of Anaerobic Liquid Digestates Through Membrane Processing and Struvite Recovery—The Case of Dairy Effluents
Source: Membranes (Basel). 2025 Jun 24;15(7):189. doi: 10.3390/membranes15070189 (PMC12300007; doi:10.3390/membranes15070189)
Supplement: Supplementary file 1 [file membranes-15-00189-s001.zip › membranes-3649305-supplementary.pdf]

Supplementary Material

"Valorization of Anaerobic Liquid Digestates Through Membrane Processing and Struvite Recovery—The Case of Dairy Effluents" by A.C. Karanasiou, C.K. Tsaridou, D.C. Sioutopoulos, C. Tzioumaklis, N. Patsikas, S.I. Patsios, K.V. Plakas, A.J. Karabelas

S1. Biogas plants in Europe

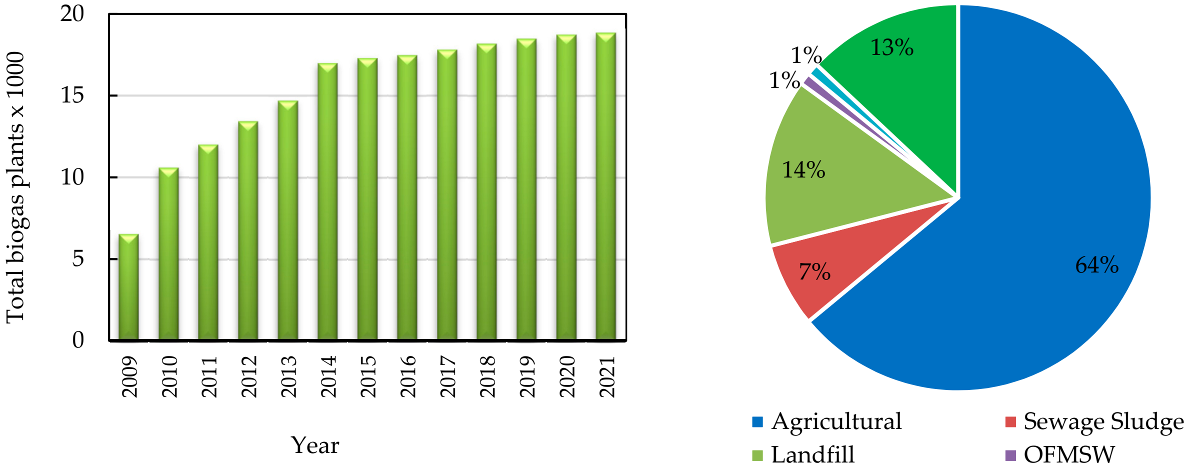

(a) (b)

Figure S1-1. (**a**) Number of biogas plants, (**b**) origin of biogas feedstocks; EU-27 and United Kingdom, Norway, Switzerland, and Iceland, 2021. OFMSW: organic fraction of municipal solid waste [1].

S2. Physico-chemical characteristics of the FLD samples (UF—permeate of the anaerobic digestate)

The composition of FLD in different seasons (i.e., autumn, winter, spring) is presented in **Table S2-1**.

Table S2-1. Physico-chemical characteristics of the FLD (UF—permeate) samples, as received from the operating industrial AD plant[1].

Sample	FLD-I	FLD-II	FLD-III	FLD-IV	FLD-V
**Months of operation of AD plant**	13[2]	13.5	16.5	17.5	20
**Month**	October	October	January	February	May
**pH**	7.3	NA	7.4	7.4	7.1
**eC, μS/cm**	6330	6140	8260	6970	8330
**Mg^{2+}, mg/L**	ND	12	20	20	25
**NH_4-N, mg/L**	188	98	213	239	268
**PO_4-P, mg/L**	101	59	102	94.3	126
**Cl^-, mg/L**	626	295	635	679	791
**SO_4^{2-}, mg/L**	ND	70	91	86.2	152
**Na^+, mg/L**	1398	1609	1758	1280	1657
**K^+, mg/L**	241	237	231	211	294
**Ca^{2+}, mg/L**	20	22	18	<20	<40
**Alkalinity, mg $CaCO_3$/L**	2364	2418	3710	2717	3703
**TOC, mg/L**	24	18	151	143.3	24

[1] Samples before any pre-treatment (acidification). [2] Former samples are presented in [2]. ND, not detected; NA, not analyzed

S3. Experimental set-ups, protocols, and conditions

S3-1. Cleaning protocols after NF pilot tests

Three cleaning protocols were employed after each *NF pilot test*, which are the following:

A. Flushing with NF permeate and tap water until restoration of the electrical conductivity (eC) and the pH of feed and the permeate.
B. Mild chemical Cleaning in Place (CIP) with alkaline solution (NaOH 0.1% by weight) followed by acid solution (HCl 0.2% by weight) and flashing with tap water. The chemical cleaning was conducted as follows:
   - Recirculation of the cleaning solution with a low flow rate (10L/min) for 10-15 min.
   - Stopping of the flow and soaking of the membranes for 10-15 min.
   - Recirculation of the cleaning solution with a high flow rate (20-25L/min) for 30 min at low pressure in order to avoid permeation.

After the chemical cleaning, the CIP vessels were emptied, and the pilot unit was flushed with tap water until the restoration of pH and eC.

C. Intensive CIP with alkaline solution (NaOH 0.1% by weight) followed by acid solution (HCl 0.2% by weight) and flashing with tap water. The chemical cleaning was conducted as follows:
   - Recirculation of the cleaning solution with a low flow rate (10L/min) for 25 min.
   - Stopping of the flow and soaking of the membranes for 2-3 h.
   - Recirculation of the cleaning solution with a high flow rate (20-25L/min) for 1 h at low pressure in order to avoid permeation.

After the chemical cleaning, the CIP vessels were emptied, and the pilot unit was flushed with tap water until the restoration of pH and eC.

Between the tests, the membranes were preserved by filling the pressure vessels with sodium bisulfite solution 0.5% w/w. **Figure S3-1** shows the flow diagram monitor of the pilot unit during acid (a) and alkaline chemical cleaning (b).

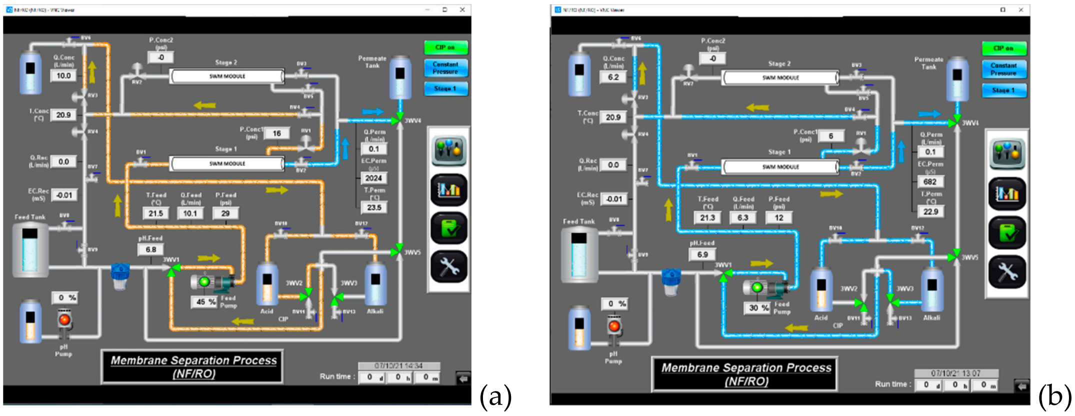

Figure S3-1. Flow diagram monitor of the pilot unit during (a) acid and (b) alkaline CIP.

S3-2. Experimental set-up for struvite precipitation

The JarTest method is commonly used for the simulation of water treatment processes and the determination of the optimal precipitation conditions, such as the amount of chemical reagents, the stirring rate and duration, the precipitation time, etc. The experimental set-up (**Figure S3-2**) comprises four stainless steel agitators controlled via an analog speed regulator with a maximum stirring rate of 300 rpm. Each stirring bar consists of a flat blade 7 cm in diameter, enabling the use of 1 L glass beakers. By these means, simultaneous experiments could be conducted by adjusting the stirring time from the integrated analog timer.

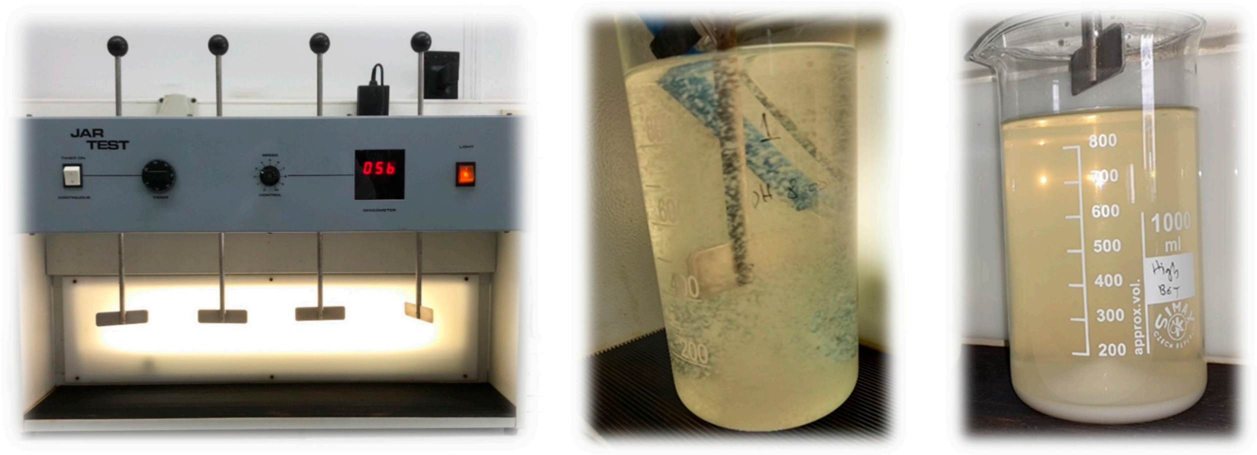

(a) (b) (c)

Figure S3-2. (**a**) Experimental set-up for the JarTests of the present study. Precipitation tests (**b**) during agitation and (**c**) 5 min after agitation.

S3-3. Struvite precipitation protocol

The struvite precipitation protocol was as follows. *(a)* A 1 L beaker was filled with 900 mL of NFC-6; *(b)* stock solutions of K_2HPO_4 (58.7% w/v) and $MgCl_2 6H_2O$ (68.3% w/v) were gradually added under constant stirring at 200 rpm for a period of 5 min (rapid stirring period); *(c)* the pH was then adjusted to the predetermined value using a 5N NaOH solution (in tests where MgO was used, this step was omitted); *(d)* the stirring rate was adjusted to 45 rpm for 10 min to aggregate the precipitate (slow stirring period); *(e)* the precipitate was allowed to settle for 30 min; *(f)* the mixture was filtered through a 1. 5μm glass fiber filter after 24 h; *(g)* finally, the precipitate was dried for 48 h in an oven or at ambient temperature. The experimental conditions of the *precipitation tests* are presented in **Table S3-1**.

Table S3-1. Experimental conditions of struvite precipitation bench-scale tests.

Test No	NH_4:Mg:PO_4 molar ratio in the feed solution	Mg source	pH	Drying temperature, °C
**SP-1**	1:1:1	$MgCl_2{\cdot}6H_2O$	8	25
**SP-2**	1:1:1	$MgCl_2{\cdot}6H_2O$	9	25
**SP-3_25**[1]				25
**SP-3_40**	1:1:1	$MgCl_2{\cdot}6H_2O$	10	40
**SP-3_105**				105
**SP-4**	1:1:1	$MgCl_2{\cdot}6H_2O$	11	25
**SP-5**	1:1:0.5	$MgCl_2{\cdot}6H_2O$	10	105
**SP-6**	1:1:1.5	$MgCl_2{\cdot}6H_2O$	10	25
**SP-7**	1:1.5:1.5	$MgCl_2{\cdot}6H_2O$	10	25
**SP-8**	1:2:2	$MgCl_2{\cdot}6H_2O$	10	25
**SP-9**	1:1.5:1.5	MgO	10	25
**SP-10**	1:1.5:1.5	MgO	9	25
**SP-11**	1:1.5:1.5	MgO-C1	9	25
**SP-12**	1:1.5:1.5	MgO-C2	9	25
**SP-13_100L**	1:1.5:1.5	$MgCl_2{\cdot}6H_2O$	10	25

[1] Drying temperature of the precipitate; MgO: analytical grade MgO; MgO-C1: conventionally produced commercial grade with low specific surface area; MgO-C2: commercial grade with high specific surface area.

S3-4. NF pilot testing conditions

The NF pilot testing conditions are listed in **Table S3-2**.

Table S3-2. NF pilot tests' conditions; Qin=~15L/min with AFLD.

Test No	Type of Test	Initial Volume, L	Cleaning Protocol*	Duration, min	P_{in}/P_{out}, bar	Q_{perm}, L/min	J, L/m²h	Recovery, %
**Pilot-1_ NFOT**	Pilot NF once-through	500	Fresh membrane	64	7.2 / 5.9	2	22.6	-
**Pilot-2_ NFOT**	Pilot NF once-through	1000	CIP	257	8.9 / 7.6	2-1.7	23.0-19.4	-
**Pilot-3_ NFBM**	Pilot NF batch mode	500	CIP	184	7.5 / 6.3	1.9-0.8	21.8-9.1	50
**Pilot-4_ NFBM**	Pilot NF batch mode	1000	Flushing	330	9.9 / 8.7	2.0-0.6	22.9-6.9	46
**Pilot-5_ NFBM**	Pilot NF batch mode	250	Intensive CIP	102	9.0-11.0 / 7.8-9.8	2.0-0.9	23.0-10.4	57
**Pilot-6_ NFBM-SP**	Pilot NF batch–struvite precipitation	250	Intensive CIP	102	10.5 / 9.3	2-0.8	23.1-9.2	56
**Pilot-7_ NFBM-SP**	Pilot NF batch–struvite precipitation	500	Intensive CIP	226	11.0 / 9.8	2.0-0.4	21.7-4.6	56

* which preceded the test; P_{in}: input pressure; P_{out}: output pressure; Q_{perm}: permeate flow rate.

S4. Experimental data/results

S4-1. Nanofiltration pilot tests

The concentration and rejection evolution during test No Pilot-2_NFOT is shown in **Figure S4-1**.

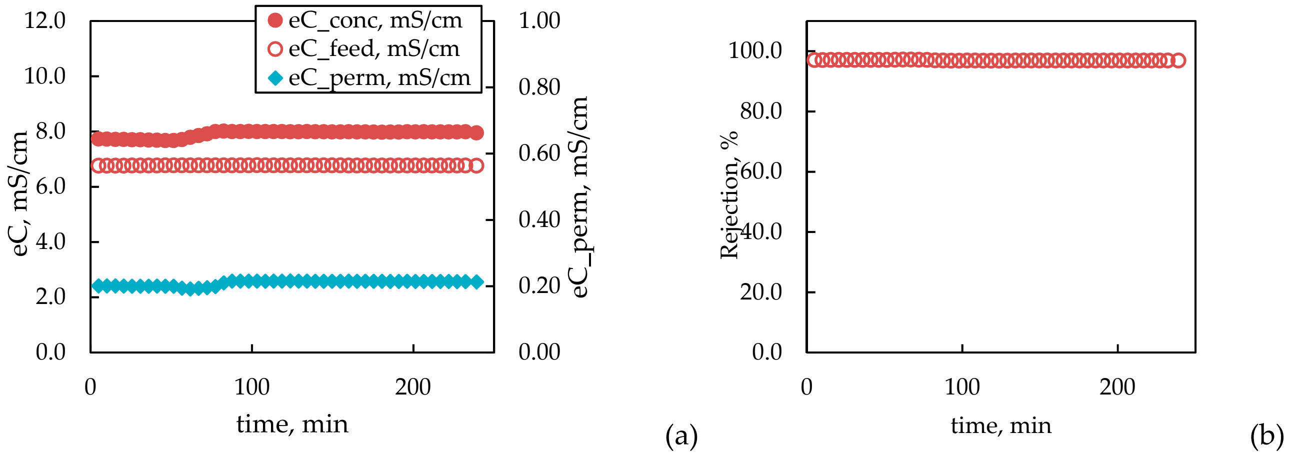

Figure S4-1. (**a**) Concentration and (**b**) rejection evolution during test No Pilot-2_NFOT.

Effluent data from the **once-through** NF pilot tests are presented in **Table S4-1**.

Table S4-1. Physico-chemical characteristics of feed, concentrate, and permeate and operating conditions from NF pilot tests; *once-through mode*, one stage (two elements), and inlet flow 15L/min.

	Pilot-1_NFOT				Pilot-2_NFOT			
**Parameter**	**Feed AFLD-1**	**Concentrate NFC-1**	**Permeate NFP-1**	**%, Rejection**	**Feed AFLD-2**	**Concentrate NFC-2**	**Permeate NFP-2**	**%, Rejection**
**pH**	6.6	N/A	N/A	-	6.7	N/A	N/A	-
**eC, μS/cm**	6282	7171	273	95.7	6958	7893	209.7	97
**Mg^{2+}, mg/L**	ND	20	ND	-	18	17	ND	-
**NH_4-N, mg/L**	173	195	7.5	95.7	123	145	4.6	96.3
**PO_4-P, mg/L**	105	115	2.5	97.6	67	64	1.2	98.2
**Cl^-, mg/L**	641	705	18	97.2	400	458	1.9	99.5
**SO_4^{2-}, mg/L**	634	705	ND	-	394	433	3.3	99.2
**Na^+, mg/L**	1467	1622	81	94.5	1512	1695	44	97.1
**K^+, mg/L**	244	278	11	95.3	250	270	7	97.3
**Ca^{2+}, mg/L**	21	31	ND	-	49	16	ND	-
**Alkalinity, mg $CaCO_3$/L**	1855	2084	NA	-	2344	NA	NA	-
**TOC, mg/L**	26	27	1.3	94.9	20.6	22	2	90.5
**Inlet pressure**	7.2 bar				8.9 bar			
**Ave. pressure**	6.5 bar				8.3 bar			
**Recovery**	13 % per pass				13 % per pass			

AFLD, acidified FLD; ND, not detected; N/A, not analyzed.

Effluent data from the **batch** NF pilot tests are presented in **Table S4-2**.

Table S4-2. Initial feed composition (acidified) for batch nanofiltration pilot tests

Test No	Pilot-3_NFBM	Pilot-4_NFBM	Pilot-5_NFBM	Pilot-6_NFBM-SP	Pilot-7_NFBM-SP
**pH**	6.9	6.8	6.8	6.8	7.0
**eC, μS/cm**	6330	7070	7530	8250	8170
**Mg^{2+}, mg/L**	NA	15	64	ND	34
**NH_4-N, mg/L**	NA	119	159	242	262
**PO_4-P, mg/L**	NA	58	109	106	118
**Cl^-, mg/L**	NA	390	784	662	997
**SO_4^{2-}, mg/L**	NA	381	953	1401	599
**Na^+, mg/L**	NA	1521	1960	1984	1942
**K^+, mg/L**	NA	235	280	254	258
**Ca^{2+}, mg/L**	NA	22	67	ND	ND
**Alkalinity, mg $CaCO_3$/L**	1880	2344	2546	2408	3283

NA: not analyzed; ND: not detected

S4-2. Struvite precipitation tests

S4-2.1. Data on the effect of pH (magnesium source: $MgCl_2$)

Figure S4-2 depicts the removal efficiency of struvite ion components (i.e., NH_4^+ ,Mg^{2+}, and PO_4^3) due to precipitation, based on Equation (2) of the paper, using the species concentration in the feed solution (after addition of the Mg and PO_4 reagents) and in the filtered supernatant solution SPF, 24 h after the start of precipitation. The effect of pH on struvite recovery appears to be particularly significant for the removal efficiency of magnesium ions, as the corresponding percentage increases from 80% (at pH 8) to ~97% (at pH 11). On the other hand, the removal of phosphates remains consistently high (>99%) within the pH range examined. In general, an increase in pH appears to be beneficial for the precipitation of struvite and as expected for the removal of Mg^{2+}.

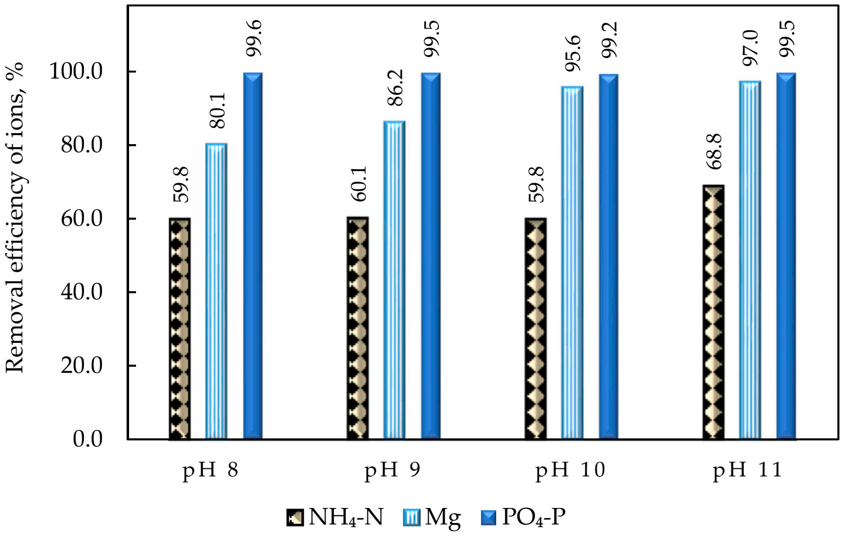

Figure 4-2. Effect of pH on the removal of struvite ionic components; Mg source: $MgCl_2{\cdot}6H_2O$, feed solution with equimolar ionic species composition, i.e. NH_4:Mg:PO_4 = 1:1:1 (Test Nos SP-1, SP-2, SP-3_25, and SP-4).

As seen in **Figure S4-3,** struvite is the only crystalline structure of the chemical precipitate. In addition, amorphous phosphate salts of Ca and Mg may have been formed, as suggested by the increased removal of these ions. Regarding ammonium removal, no significant changes were observed in most of the pH ranges studied, as removal remained nearly constant at ~60%. However, a slight increase in NH_4^+ removal (up to ~69 %) was observed at pH 11. Increasing the pH from 10 to 11 appears to have a negligible effect on Mg^{2+} removal, as this is only increased by ~1.5% (from 95.6% to 97%).

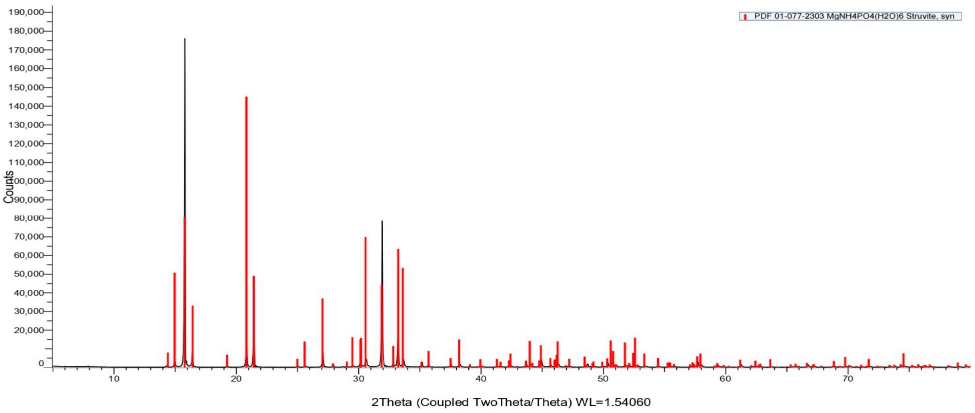

Figure S4-3. XRD spectrum of struvite. Conditions: Mg source: $MgCl_2·6H_2O$; NH_4:Mg:PO_4 = 1:1:1; pH=10; drying at ambient temperature (Test No SP-3_25).

Table S4-3 includes the concentration of struvite components in filtrated supernatant (SPF) solutions after struvite precipitation. The NH_4^+ concentration is in the range of 195-255 mg/L (TAN, Total Ammonium Nitrogen, 150-200 mg/L).

Table S4-3. Effect of pH on the concentration of struvite components in filtrated supernatant (SPF) solutions (24 h after the commencement of precipitation); NH_4:Mg:PO_4 molar ratio in the feed solution 1:1:1; Mg source: $MgCl_2·6H_2O$ (test Nos SP-1 to SP-4).

	**Feed solution**	**Feed solution after the addition of Mg^{2+} and PO_4^{3-} salts[1]**	**Filtrate (24 h) SPF 1-4**			
	**NFC-6**		**pH 8**	**pH 9**	**pH 10**	**pH 11**
**NH_4^+, mg/L**	656 ± 25	630 ± 3.9	255	252	252	195
**NH_4^+-N, mg/L**	509 ± 20	490 ± 3.0	198	196	196	151
**Mg^{2+}, mg/L**	61 ± 7	852 ± 5.3	171	119	37.8	25.0
**PO_4^{3-}, mg/L**	757 ± 40	3330 ± 21	12.9	<15.3	27.0	<15.3
**PO_4^{3-}P, mg/L**	247 ± 15	1086 ± 7.1	4.2	<5	8.8	<5

[1] The concentration is lower compared to NFC due to dilution attributed to the addition of the NaOH, $MgCl_2·6H_2O$, and K_2HPO_4 for pH and NH4:Mg:PO4 molar ratio adjustment.

S4-2.2. Data on the duration of the struvite precipitation process (magnesium source: $MgCl_2$)

Table S4-4. Concentrations of struvite components in the initial and final solutions of the precipitation tests performed at pH=10, NH_4:Mg:PO_4 molar ratio in the feed solution 1:1:1, Mg source: $MgCl_2·6H_2O$ (test No SP-4).

	**Feed solution NFC-6**	**Feed solution after the addition of Mg^{2+} and PO_4^{3-} salts[1]**	**Supernatant (*24h*) SPS-4_*24h***	**Filtrate (*24h*) SPF-4_*24h***
**NH_4^+, mg/L**	656 ± 25	628	269	252
**NH_4^+-N, mg/L**	509 ± 20	489	209	196
**Mg^{2+}, mg/L**	61 ± 7	850	39.2	37.8
**PO_4^{3-}, mg/L**	757 ± 40	3323	<15.3	27
**PO_4^{3-}-P, mg/L**	247 ± 15	1083	<5	8.8
	**Feed solution NFC-6**	**Feed solution after the addition of Mg^{2+} and PO_4^{3-} salts[1]**	**Supernatant (30min) SPS-4_30min**	**Filtrate (30 min) SPF-4_30min**
**NH_4^+, mg/L**	656 ± 25	606	242	216
**NH_4^+-N, mg/L**	509 ± 20	471	188	168
**Mg^{2+}, mg/L**	61 ± 7	850	132	107
**PO_4^{3-}, mg/L**	757 ± 40	3317	16.9	13.8
**PO_4^{3-}-P, mg/L**	247 ± 15	1081	5.5	4.5

[1] The concentration is lower compared to NFC due to dilution attributed to the addition of the NaOH, $MgCl_2·6H_2O$, and K_2HPO_4 for pH and NH4:Mg:PO4 molar ratio adjustment.

S4-2.3. Data on the effect of the NH_4:Mg:PO_4 molar ratio (magnesium source: $MgCl_2$)

Table S4-5. Effect of NH_4:Mg:PO_4 *molar ratio* on the concentrations of struvite components before and after 30 min of precipitation; pH=10, precipitation time=30 min, Mg source: $MgCl_2 \cdot 6H_2O$ (test Nos SP-3 and SP5 to SP-8).

	**NH_4:Mg:PO_4 molar ratio in the feed solution**					
	**Feed**	**Supernatant (SPS_30min)**				
	**solution**	**1:1:0.5**	**1:1:1**	**1:1:1.5**	**1:1.5:1.5**	**1:2:2**
	NFC-6	**SPS-5**	**SPS-3**	**SPS-6**	**SPS-7**	**SPS-8**
**NH_4^+, mg/L**	656 ± 25	464	242	231	154	47.6
**NH_4^+-N, mg/L**	509 ± 20	360	188	180	120	37.0
**Mg^{2+}, mg/L**	61 ± 7	163	132	13.8	62.0	129
**PO_4^{3-}, mg/L**	757 ± 40	10.7	16.9	838	23.9	446
**PO_4^{3-}-P, mg/L**	247 ± 15	3.50	5.5	273	7.80	146

[1] Average value of all of the experiments

In **Figure S4-4,** typical SEM images of the precipitates are included for precipitation using $MgCl_2 \cdot 6H_2O$ as a Mg source at various NH_4:Mg:PO_4 molar ratios, indicating the structure of struvite.

NH4:Mg:PO4 1:1:1 Test No SP-3 | NH4:Mg:PO4 1:1:1.5 Test No SP-6 | NH4:Mg:PO4 1:1.5:1.5 Test No SP-7 | NH4:Mg:PO4 1:2:2 Test No SP-8

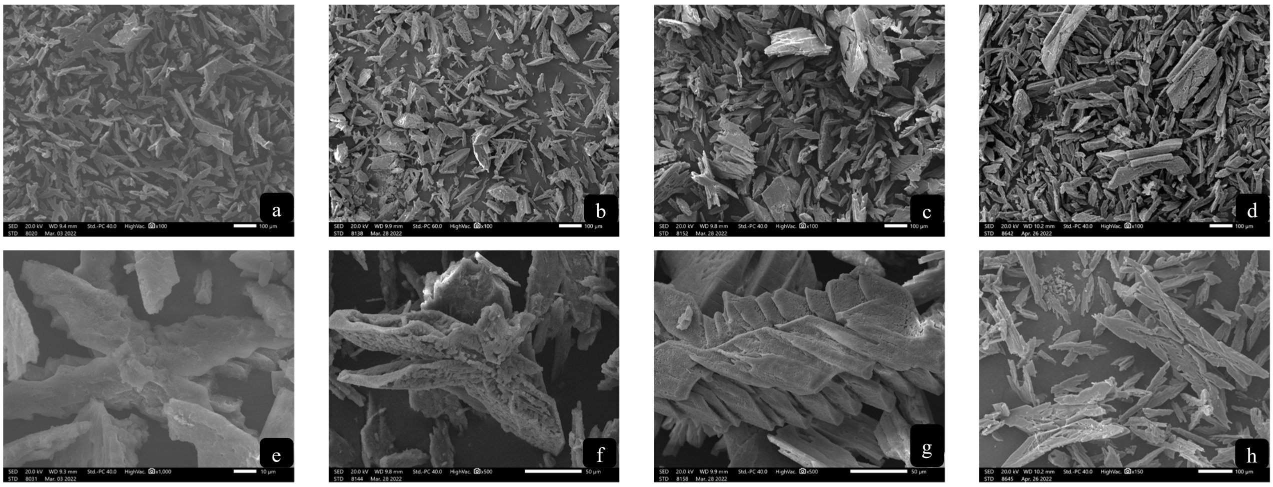

Figure S4-4. SEM images of precipitate samples from struvite precipitation tests at various NH_4:Mg:PO_4 molar ratios (a-d) x100, (e) x1000, (f-g) x500, and h) x150; Mg source $MgCl_2 \cdot 6H_2O$, pH=10, precipitation time=30 min, drying at ambient temperature.

In **Figure S4-5,** the XRD spectra of the precipitates are depicted for precipitation using $MgCl_2 \cdot 6H_2O$ as an Mg source at various NH_4:Mg:PO_4 molar ratios, revealing the chemical composition and crystal structure of struvite.

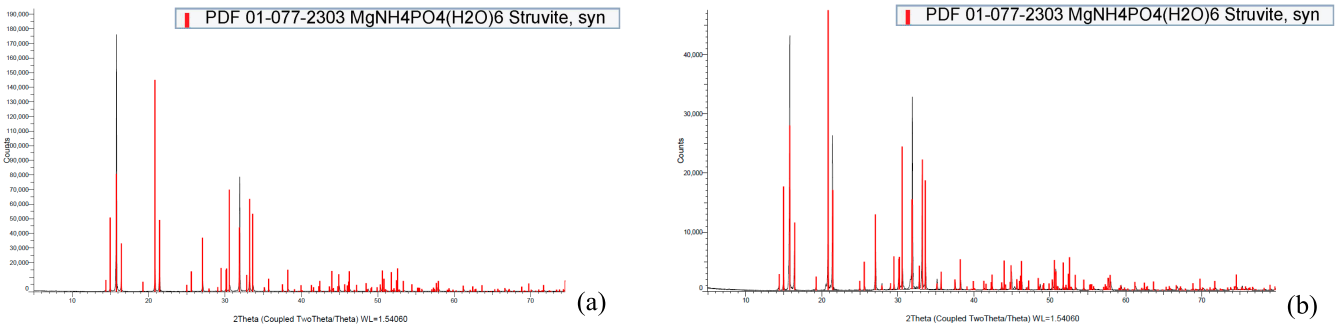

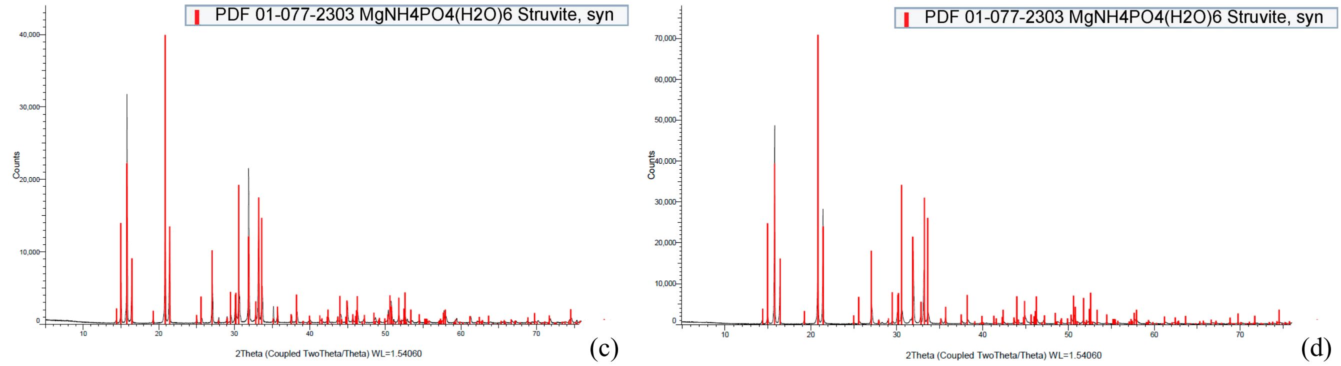

Figure S4-5. XRD spectrum of precipitate samples from struvite precipitation tests; NH_4:Mg:PO_4 molar ratios in feed solution: a) 1:1:1 (SP-3), b) 1:1:1.5 (SP-6), c) 1:1.5:1.5 (SP-7), and d) 1:2:2 (SP-8); Mg source $MgCl_2{\cdot}6H_2O$, pH=10, precipitation time=30 min, drying at ambient temperature.

S4-2.4. Data on the effect of the drying temperature on the precipitate's structure (magnesium source: $MgCl_2$)

The separated solid precipitate (test No SP-3: equimolar ratio 1:1:1, pH 10) was dried at three different temperatures, namely, room temperature (~25°C), 40°C, and 105 °C, and the moisture content was determined based on the weight difference of the precipitate before and after the drying process. As seen in **Table S4-6**, increasing the drying temperature resulted in a significant decrease in the weight of the solid precipitate. Specifically, at the highest drying temperature (i.e., 105°C), a 73.4% decrease in the weight of the precipitate was observed, while a decrease of 51.2% and 48.1% was observed when drying at 40°C and 25°C, respectively. After dissolution of the dry precipitate in HCl solution, the concentration and molar ratio of struvite ion components were also determined to obtain information on the structure of the precipitate. The data in **Table S4-6** show that at lower drying temperatures (25°C and 40°C), the solid precipitate consists of an almost equimolar concentration of NH_4, Mg, and PO_4, while at the higher temperature (105°C) the ammonium ions are significantly reduced. Additionally, struvite's purity is significantly lower in the precipitate dried at 105°C.

Table S4-6. Weight loss (%) due to drying of the precipitate and composition of the dry precipitate after drying at different temperatures (test No SP-3).

Drying temperature	25 °C	40 °C	105 °C
% weight reduction	48.1	51.2	74.3
NH_4-N, % w/w	4.4	4.3	1.0
Mg, % w/w	11	11	20
PO_4-P, % w/w	13	13	23
Struvite's purity (%)[1]	76.9	75.2	17.5
NH_4:Mg:PO_4 in the dry precipitate	0.7:1:0.9	0.7:1:0.9	0.1:1:0.9

[1] Equation (3) of the manuscript

The previous findings cannot be clearly identified in the SEM images of the three precipitates after drying (**Figure S4-6**). However, the crystalline species formed during the drying process under mild conditions seem to be in the form of well-developed crystalline deposits, while the higher drying temperature is associated with the formation of more amorphous deposits.

SP-3_25 SP-3_40 SP-3_105

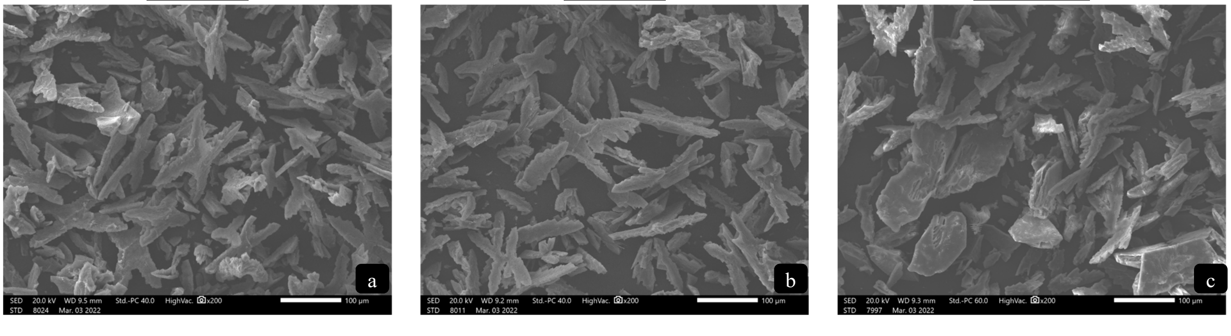

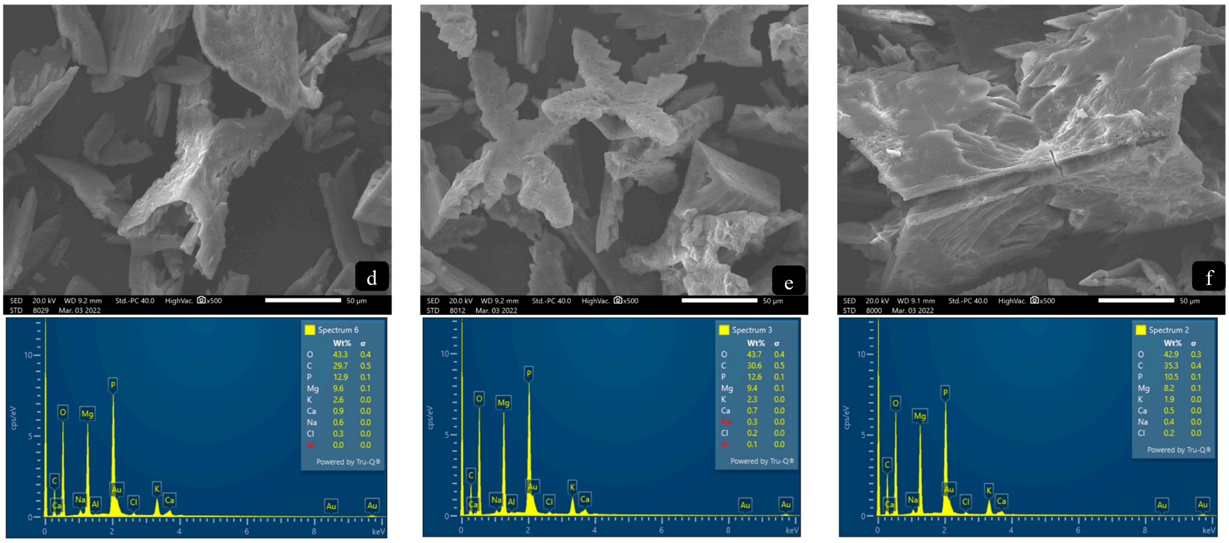

Figure S4-6. SEM images and EDS spectra of precipitate samples from struvite precipitation tests dried at various temperatures, (a-c) x200 and (d-f) x500; Mg source $MgCl_2{\cdot}6H_2O$, pH=10, NH_4:Mg:PO_4 1:1:1, precipitation time=30 min (test No SP-3).

The crystalline form of struvite was also easily detected through XRD analyses of samples dried at ~25 and 40 °C (**Figure S4-7**). On the contrary, the XRD spectrum after the drying process at 105 °C exhibits the pattern encountered in amorphous materials or mixtures. Therefore, it can be concluded that a drying process at temperatures up to 40°C should be selected for the struvite precipitate to avoid degradation of its structure.

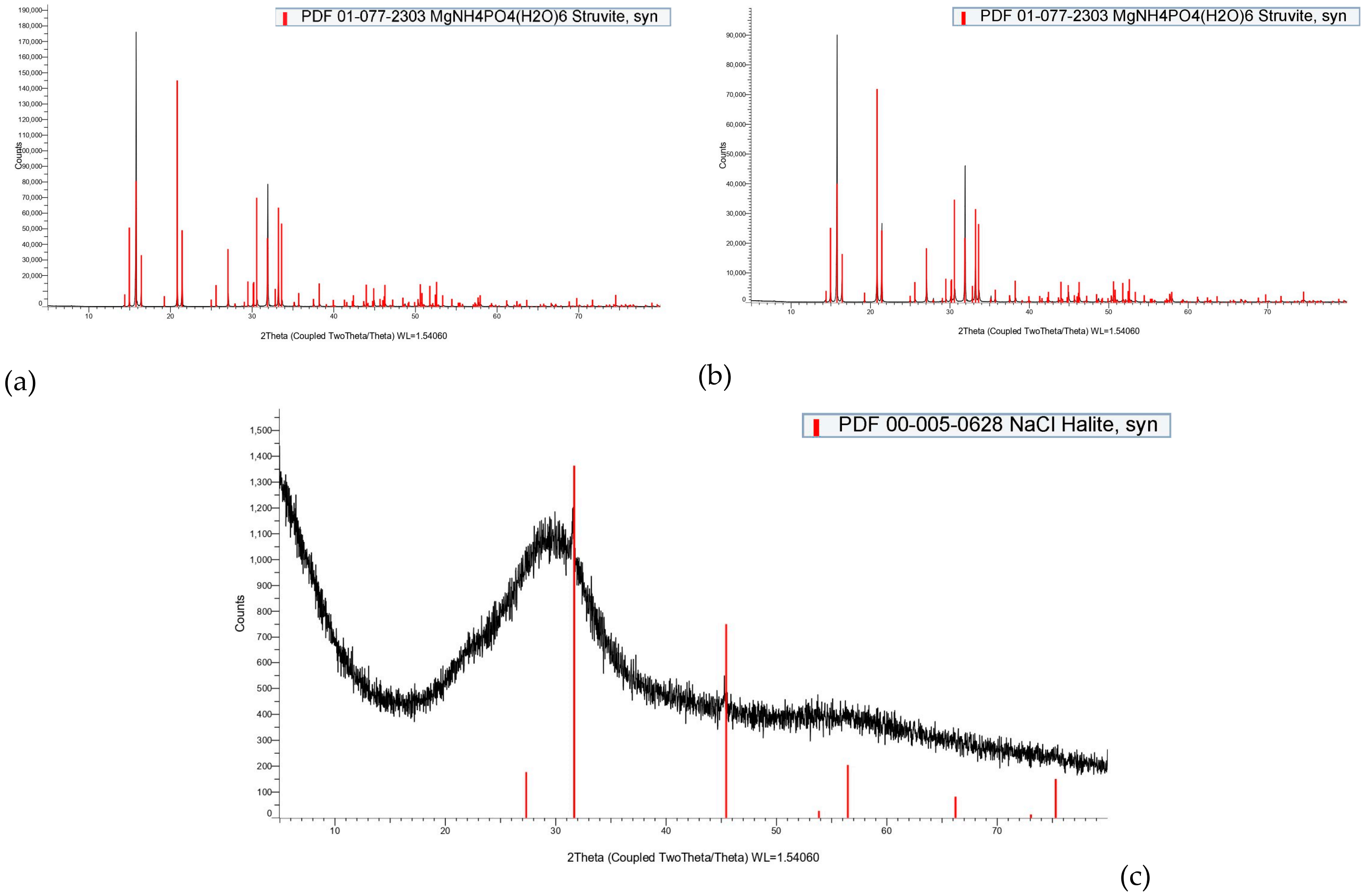

Figure S4-7. XRD spectrum of the precipitate dried at (a) ambient temperature (25 °C), (b) 40 °C, and (c) 105 °C; Mg source $MgCl_2{\cdot}6H_2O$, pH=10, NH_4:Mg:PO_4 1:1:1, precipitation time=30 min (test No SP-3).

S4-2.5. MgO used as a magnesium source

Table S4-7. Effect of *Mg source* on the concentrations of struvite components before and after 30 min precipitation; precipitation time=30 min, **NH_4:Mg:PO_4** 1:1.5:1.5 (test Nos SP-5 and SP-9 to SP-12)

	**NH_4:Mg:PO_4 molar ratio in the feed solution**					
	**Feed solution**	**Supernatant SPS_30min**				
	NFC-6	$MgCl_2$[1]	MgO[1]	MgO[2]	MgO-C1[2]	MgO-C2[2]
**NH_4^+, mg/L**	686 ± 4.7	154	366	362	206	276
**NH_4^+-N, mg/L**	532 ± 2.9	120	285	310	160	215
**Mg^{2+}, mg/L**	60 ± 2.5	62.0	ND	33	ND	20.5
**PO_4^{3-}, mg/L**	718 ± 12.2	23.9	1681	1908	1030	2360
**PO_4^{3-}-P, mg/L**	234 ± 4.0	7.80	549	623	336	770

[1] pH=10, [2] no pH adjustment (pH~9); ND, not detected

Table S4-8. *Effect of* Mg source on mass and nutrient concentration in the dry precipitate; dried at ambient temperature, **NH_4:Mg:PO_4** 1:1.5:1.5 (test Nos SP-5 and SP-9 to SP-12)

**Mg source**	$MgCl_2$	MgO	MgO	MgO-C1	MgO-C2
**pH**	10	10	9	9	9
**Mass of dry precipitate per feed volume, g/L**	11.8	7.8	7.4	10.5	8.9
**NH_4-N, % w/w^{db}**	2.9	2.3	2.3	4.1	4.1
**Mg, % w/w^{db}**	10.7	17.8	18.1	12.9	13.0
**PO_4-P, % w/w^{db}**	11.8	5.5	5.7	5.8	6.3
**NH_4:Mg:PO_4 in precipitate^{db}**	1:2.1:1.1	1:4.5:1.1	1:5.1:1.1	1:1.8:0.6	1:1.6:0.7

^{db} dry basis

References

1. European Biogas Association, *EBA Statistical Report 2022: Tracking biogas and biomethane deployment across Europe*, Brussels, 2022, Available online: https://www.europeanbiogas.eu/__trashed-3/ (accessed on 24 March 2023).
2. Tsaridou, C.; Karanasiou, A.; Plakas, K.V.; Karabelas, A.J. Valorization of Anaerobic-Fermentation Liquid Digestates - Membrane-Based Process Development. *Membranes* **2023**, *13*, 297, doi:https://doi.org/10.3390/membranes13030297.
